# Graph pan-genome advances genetic discoveries and the improvement of eggplant

**DOI:** 10.1093/hr/uhaf248

**Published:** 2025-09-19

**Authors:** Chuying Yu, Weiliu Li, Yaqin Jiang, Qihong Yang, Guiyun Gan, Liangyu Cai, Wenjia Li, Yikui Wang

**Affiliations:** Vegetable Research Institute, Guangxi Academy of Agricultural Sciences, Nanning 530007, China; Vegetable Research Institute, Guangxi Academy of Agricultural Sciences, Nanning 530007, China; Vegetable Research Institute, Guangxi Academy of Agricultural Sciences, Nanning 530007, China; Vegetable Research Institute, Guangxi Academy of Agricultural Sciences, Nanning 530007, China; Vegetable Research Institute, Guangxi Academy of Agricultural Sciences, Nanning 530007, China; Vegetable Research Institute, Guangxi Academy of Agricultural Sciences, Nanning 530007, China

## Abstract

Eggplant is one of the most important solanaceous vegetable crops worldwide. To explore its genomic diversity, we assembled two T2T-level reference genomes from the African eggplant ‘Y11’ (*Solanum aethiopicum* L.) and the cultivated variety ‘Gui5’ (*Solanum melongena* L.) with genome sizes of 1.10 and 1.13 Gb, respectively. The contigs N50 lengths are 94.2 and 93.9 Mb, with annotations of 37 324 and 40 300 protein-coding genes correspondingly. We also sequenced 238 germplasms, primarily local and cultivated varieties from China, Southeast Asia, Europe, and Africa, identifying 7 853 531 high-quality single nucleotide polymorphisms. Phylogenetic trees and population structures suggest that the domestication of Chinese eggplants occurred later than in Southeast Asia and subsequently diverged into northern and southern groups within China, evolving relatively independently with limited genetic flow between these two groups. Their diversity is significantly lower than that of Southeast Asia and Europe. By selecting 22 representative accessions and four chromosome-level genomes, we constructed an Asian-representative eggplant pan-genome, assembling 463.94 Mb of nonreference sequences. Of these sequences, 38.3% are core genes, 46.9% are dispensable genes, and 14.9% are unique genes. Presence/absence variation genes were found to be highly associated with stress resistance in eggplants. Genome-wide association studies identified 946 SNPs and 9605 genes significantly associated with 10 important traits. Notably, genes involved in zeatin biosynthesis closely linked to plant auxins significantly impact fruit size and shape attributes, playing a crucial role in eggplant yield. This high-quality reference genome alongside the pan-genome will provide valuable insights into eggplant breeding advancement.

## Introduction

Eggplant (*Solanum melongena* L., 2*n* = 24) is an annual herbaceous plant of the genus Solanum in the family Solanaceae, its immature fruit is the main product organ and has important economic value [1]. Early studies suggested that the African species *S*. *incanum* spread to Asia, further domesticated in Asia as a modern cultivated eggplant [2]. Crop breeding methods have gone through different stages of development, from domestication to selection. The main early breeding method used the excellent traits of wild germplasm to carry out domestication and selection [3]. With the advent of Mendelian inheritance, artificial selection through cross breeding, such as dwarf breeding of wheat and corn [4]. During the long-term domestication, selection, and cultivation of wild Solanaceae plants, their yields and commodity quality have been continuously improved driven by altered cultivation environments and people’s consumption preferences. This focus has simultaneously led to a decline in resistance to biotic and abiotic stresses, along with the loss of certain functional components. Consequently, biologists and breeders seek to identify valuable traits or genes from wild germplasm to overcome genetic improvement bottlenecks in related crops. As a wild relative of cultivated eggplant (*S*. *melongena*), the African eggplant (*S*. *aethiopicum*) is consumed in regions such as Africa and China (e.g. Guangxi, Yunnan). Hybridization enables the introgression of its beneficial traits to enhance stress resistance in cultivated eggplant, with heterosis observed in some interspecific *Solanum* hybrids [5, 6]. Later the gradual development of molecular biology subsequently enabled the application of genetic engineering technology in crop breeding. The application of marker-assisted selection (MAS) greatly shortened the breeding period, accelerated the crop breeding process, and became an efficient breeding method [7, 8]. Nevertheless, the absence of a high-quality reference genome impedes adequate resolution of the genetic differences between *S. aethiopicum* and *S. melongen*a, thereby challenging the localization of structural variants (e.g. large indels) linked to specific traits.

In recent years, with the rapid development of third-generation sequencing technology, more complex plant genomes have been assembled and optimized, and more new genes have been discovered, marking a new era in plant genome research, which has greatly promoted genome-guided crop breeding [9]. The construction of high-quality reference genomes is the basis for multiomics research and molecular breeding (such as genomic selection (GS) and MAS) [10]. Since the beginning of this century, plant genome sequencing has been carried out rapidly, and the quantity and quality of genomic resources have significantly improved. At present, the genome sequencing of nearly 800 plant species and subspecies has been completed [11]. Owing to the slow assembly of the eggplant genome, the scaffold version of the genome sequence of S. Nakate-Shinkuro eggplant was not completed until 2014 and yielded a fragmented result (contig N50 < 1 Mb). Hundreds of assembly gaps clustered within repetitive sequence-rich centromeres resulted in the loss of critical regulatory element annotations [12]. In 2019, the sequencing of African red eggplant (*S. aethiopicum*) was completed [13]. The genome sequencing and assembly of the European type eggplant 67/3 was completed using the second-generation sequencing technology, and a chromosome-level genome was reassembled using Hi–C technology in 2021 [14, 15]. Wei *et al.* used ‘Hangqie-1315’ cultivar and third-generation ONT sequencing technology to obtain an eggplant genome with a contig N50 of 5.3 Mb [16]. Li *et al.* sequenced and assembled the ‘GUIQIE-1’ genome with a contig N50 size of 5.3 Mb [17]. With the successful assembly and sequencing of these eggplant genomes, research on the eggplant genome has entered the ‘high-definition era’. Even so, eggplant genomics research still faces a series of challenges. The genome of the eggplant, characterized by occurrences of whole genome duplication (WGD) and a substantial prevalence of repetitive sequences, presents considerable obstacles in the assembly of specific regions such as centromeres, telomeres, and ribosomal DNA. Despite the availability of a comparatively high-quality reference genome for cultivated eggplant [18], there persists an absence of an equally refined reference genome for wild eggplant species. With the advancements in third-generation sequencing technology, especially the combination of ultralong Oxford Nanopore Technologies (ONT) ultralong sequencing and high-accuracy PacBio high-fidelity long-read (HiFi) sequencing, the difficulties in assembling centromeric and highly repetitive regions of the genome have been overcome. With the development of sequencing technology, the complete T2T genome has been successfully assembled, and gaps in the previous genome have been filled [19]. The assembly of the T2T genomes of different species has been completed one after another [20–24]. On the basis of these findings, the annotation of a series of important genes has not only promoted the historical process of plant functional genomics research but also provided ideas for genome-wide association study (GWAS) of important traits through resequencing and will help in the genetic analysis of important traits. In addition, GWAS has been used to analyze the formation mechanisms of a number of important agronomic traits in crops such as wheat, maize, rice, tomato and cotton, providing opportunities for the discovery and development of candidate genes [25–29].

The rapid development of sequencing technology has made crop evolution and geographical origin, gene regulation, structural variation (SV), genetic linkage mapping, and population evolution analysis hot topics. Moreover, a single representative genome cannot represent all the genetic information of a species, and some sequences and genetic variations missing from the reference genome are lost [30]. Many subpopulation-specific genetic variations cannot be found in the reference genome, and this missing information is likely to be correlated with traits [31]. Therefore, the construction of nonreference sequences that do not appear in the reference genome and the existing reference genome to form a pan-genome can contain all the genetic variations of a species, which helps to fully analyze the relationships between the traits and genes of the species [32]. The concept of the pan-genome emerged, and crop genome research entered the pan-genome era. Since the successful construction of the first soybean pan-genome in 2014, pan-genome maps of crops such as rice, maize, wheat, and cotton have also been published [33–37]. These studies have discussed the close relationship between genome SVs and gene expression and have made substantial contributions to important phenotypic variations. The concept of the pan-genome provides a new idea for the study of genetic diversity between and within species. When comparative genomic studies on eggplant are conducted, the pan-genome can capture the genetic variation within a species more comprehensively and accurately, thus providing a powerful tool for gaining a deeper understanding of the structure and evolution of the eggplant genome.

With the development of breeding technology and artificial selection, eggplants are gradually bred toward high yield and high quality, and their varieties are becoming increasingly homogeneous, resulting in a narrowing of genetic resources [38]. Wild germplasm resources are the original form of plant populations in the natural environment, in the long-term natural selection process, these plants present strong disease resistance and stress resistance, are rich in diverse genetic backgrounds, and retain many high-quality genes, these genes are valuable resources for improving and breeding high-quality cultivated varieties [39]. The T2T genome further improves the accuracy of genetic variation research and can be used as a stable pan-genome skeleton to construct a pan-genome for the exploration of SV. At the same time, it can also lay the foundation for the analysis of the formation mechanism of important agronomic traits and the selection of varieties that meet breeding goals [24]. However, research on the mechanism of important eggplant traits is relatively rare, and many formation and regulatory mechanisms of traits still need to be analyzed. To this end, this study assembled high-quality T2T genomes of the African eggplant ‘Y11’ and cultivated cultivar ‘Gui5’ through HiFi, ONT, and Hi-C data and resequenced 238 eggplant germplasms from China, Southeast Asia, Europe, and Africa, identifying 7.8 million high-quality single-nucleotide polymorphisms (SNPs) for GWAS analysis of important eggplant traits and revealing evolutionary paths and population structures. In comparative genomic studies of eggplant, pan-genomes offer an enhanced and precise encapsulation of genetic variability within species, thus providing a powerful tool for obtaining an in-depth understanding of the structure and evolution of the eggplant genome. Furthermore, 22 representative cultivars were selected to construct pan-genomes, and nonreference sequences, including core genes, dispensable genes and unique genes, were identified. By integrating the high-quality T2T genomes of wild red eggplant and cultivated eggplant with pan-genomes and population genetic information, important insights into the genetic diversity and evolution of eggplant were obtained. The identification of genes and loci associated with key traits provides valuable resources for accelerating eggplant breeding and helps to carry out molecular breeding in a comprehensive and in-depth manner. The discovery and exploration of a large number of genes of unknown function and the analysis of their molecular mechanisms will provide new insights into the historical process of eggplant genes adapting to environmental growth and continuous development and evolution.

**Figure 1:**
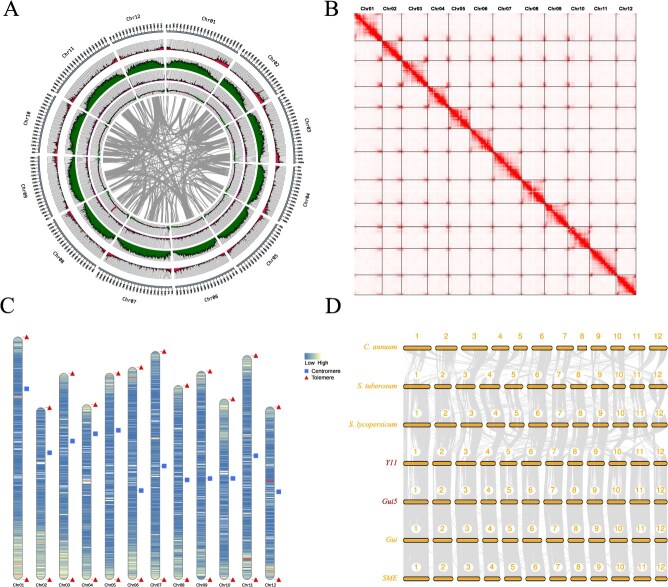
Genomic assembly and synteny analysis of the ‘Y11’ Eggplant. **A.** This figure presents a comprehensive overview of the genomic features of the ‘Y11’ eggplant. The concentric circles illustrate, from the outermost to the innermost layer: 1) gene density, assessed with a 200 kb sliding window; 2) transposable element density, similarly evaluated using a 200 kb sliding window; 3) density of repetitive sequences; 4) GC content, with values above the genome-wide average distinguished from those below it; and 5) syntenic blocks of genes. **B.** The Hi-C heat maps provide insights into the chromosomal configuration of the ‘Y11’ eggplant genome. **C.** Mapping of telomeres and centromeres on assembled chromosomes. A gradient color scale represents gene density, with numerals marking chromosome identifiers. **D.** Syntenic analysis of four eggplant varieties (‘Y11’, ‘Gui5’, ‘Gui’, ‘SME’) and three related solanaceae species (*C. annuum*, *S. tuberosum*, and *S. lycopersicum).*

## Results

### *De novo* assemblies and annotation of T2T genomes from ‘Y11’ (*S*. *aethiopicum*) and ‘Gui5’ (*S*. *melongena*)

In pursuit of superior reference genomes for the African eggplant known as ‘Y11’ and the cultivated eggplant labeled as ‘Gui5’ (Fig. S1), we harnessed the Pacific Biosciences (PacBio) HiFi sequencing technology. This cutting-edge approach yielded a substantial output of 94.80 and 79.81 Gb of HiFi data, respectively (Table S1). Initial *de novo* assembly using HiFi sequencing data yielded contigs with N50 values of 76.66 and 36.51 Mb, respectively. To elevate these genomic resources to chromosome-level accuracy, we constructed and sequenced Hi-C libraries, generating 162.75 and 140.75 Gb of reads (Table S1). Utilizing the resulting Hi–C interaction matrices, we anchored the assemblies of both samples onto twelve chromosomes each, thereby producing two near telomere-to-telomere (T2T) reference genomes with total sizes of 1.10 (Fig. 1A and B) and 1.13 Gb (Fig. S2), respectively (Table 1). To address the 18 gaps in the ‘Y11’ assembly and the 34 gaps in ‘Gui5’, thereby enhancing reference genome quality to T2T standards, we constructed additional ONT ultra-long read libraries and performed deep sequencing, generating 45.20 and 21.10 Gb of ultra-long sequence data (Table S1) for each sample, respectively. The incorporation of these long reads facilitated efficient gap closure: as a result, the assembly of ‘Y11’ was resolved into a completely contiguous, gapless T2T genome, while only a single remaining gap persisted across the entire genome of ‘Gui5’. By meticulously identifying telomeric sequences, we established that the ‘Gui5’ genome alone exhibits partial telomeric deficiency on three chromosomes (Table S2). In contrast, all other chromosomes within this genome contain discernible dual-ended telomeres. For the elucidation of centromeric sequences within these genomes, we primarily relied on the analysis of tandem repeat arrays. Our findings indicated that centromeric DNA constitutes a significant portion of the most prevalent tandem repeats (Table S3). With meticulously assembled telomeres and minimally divergent centromeric regions identified, it is evident that these two genomes exemplify outstanding standards—reflecting high-quality references akin to genuine T2T-level criteria.

**Table 1:** Comparison between newly assembled T2T eggplant genomes ‘Y11’ and ‘Gui5’ with published ‘Gui’ and ‘SME’ genomes.

**Iterms**	**Gui** ^**a**^	**SME** **^b^**	**Y11**	**Gui5**
Estimated heterozygosity rate	–	0.15%	0.08%	0.06%
Estimated size (GB)	–	1.21	1.20	1.20
Genome size (GB)	1.16	1.07	1.10	1.13
Contigs num	625	2,945	79	106
Max contig length	21.7	38.8	109.7	114.1
Contig N50 (Mb)	5.3	5.3	94.2	93.9
Scaffold num	319	2,263	12	12
Telomeres num	–	–	20	24
Max scaffold length (Mb)	112	106.8	109,7	114.1
Scaffold N50 (Mb)	93.9	89.64	94,2	93.9
Gaps num	–	–	0	1
Gaps num	–	–	0	1
Repeat percent (%)	70.1	70.1	68.9%	69.6%
BUSCOs completeness of assembly	1,440 (96.2%)	2,190 (94.2%)	1,614 (97.5%)	1,614 (97.0%)
Annotated genes num	35,018	36,582	37,324	40,300
BUSCOs completeness of annotation	1,440 (96.6%)	–	1,614 (98.4%)	1,614 (98.6%)

a The genome sequence was retrieved from the NCBI BioProject database (accession PRJNA612792) [17].

b The genome assembly could be accessed at http://eggplant-hq.cn [16].

To assess the quality and completeness of the assembled genomes, we employed four rigorous methodologies: firstly, Benchmarking Universal Single-Copy Orthologs (BUSCO) analysis revealed that both eggplant genomes achieved approximately 97.0% completeness. Secondly, alignment of HiFi and short-read data against the assemblies demonstrated remarkable alignment rates exceeding 99.8%. Thirdly, the assessment of the long terminal repeat (LTR), Assembly Index (LAI) revealed values of 14.44 for ‘Y11’, and 16.66 for ‘Gui5’, further cementing their status as superior quality genomes. Lastly, using next-generation sequencing (NGS) data, we calculated QV values for each reference genome: ‘Gui5’ exhibited a QV of 59.24 and ‘Y11’ a QV of 58.57, affirming both as high-quality, complete T2T genomes. Additionally, a synteny analysis with previously published eggplant genomes demonstrated strong collinearity among the three cultivated varieties (‘Gui5’, ‘Gui’, and ‘SME’), whereas substantial SVs were observed in comparison with the African eggplant ‘Y11’ (Fig. 1D). Collectively, these metrics underscore that both T2T genome assemblies represent high-quality genetic resources.

In order to acquire genome annotations of excellent quality, we integrated *de novo* prediction, homology-based prediction, and transcriptomic analysis. This comprehensive approach led to the identification of 37 324 and 40 300 protein-coding genes within the ‘Y11’ and ‘Gui5’ T2T reference genomes, respectively (Table 1). The average gene length was determined to be 6151 bp for ‘Y11’ and 5773 bp for ‘Gui5’, while the mean coding sequence (CDS) lengths were 1231 and 1053 bp correspondingly (Table S4). BUSCO evaluations of these protein-coding genes indicated completeness scores exceeding 98.4%, underscoring the exceptional quality of these genomic annotations (Table S5). Functional annotation revealed that 94% of ‘Gui5’s protein-coding genes had predicted functions in databases, whereas ‘Y11’ demonstrated an even higher functional prediction rate at 96% (Table S6). In terms of repetitive sequence annotation, the genomes were composed of 68.9% and 69.7% repetitive elements for ‘Y11’ and ‘Gui5’, respectively (Table S7). Among these sequences, LTR type retrotransposons were identified as the predominant components, accounting for 55.2% and 52.0% of the sequences, respectively. Notably, LINEs occupied approximately 19.2 and 22.7 Mb within the respective genomic landscapes. Furthermore, our analysis identified the presence of 1195 tRNAs and 1447 tRNAs, alongside 198 snRNAs and 215 snRNAs in the ‘Y11’ and ‘Gui5’ genomes, respectively. Additionally, both genomes contained an identical count of 325 snoRNAs (Table S8).

### Comparative genomics and adaptive insights into eggplant varieties

**Figure 2:**
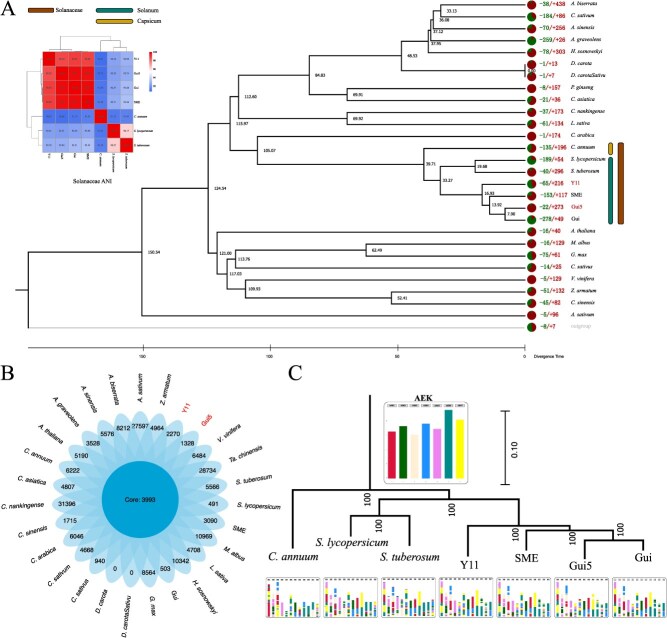
Comparative genomic analysis and ancestral chromosomal evolution. A. The evolutionary tree illustrates the phylogenetic relationships among 27 species, along with their divergence times. The number of gene families that have undergone expansion (+) or contraction (−) during evolution is depicted. In the top left corner, an average nucleotide identity (ANI) heatmap is presented, where a similarity of 98% or higher is generally indicative of the same species. B. Orthologous gene families among the 27 species were identified using OrthoFinder. The numbers refer to the gene families recognized for each species. C. Depiction of ancestral chromosomal-level genomic evolution within the Solanaceae family, including four *Solanum* varieties, *S. tuberosum*, *S. lycopersicum,* and *C. annuum.*

In a comprehensive comparative genomic analysis involving 27 species, including seven from the Solanaceae family, we examined gene families across these species. This included two newly assembled *Solanum* genomes (‘Y11’ and ‘Gui5’) from our study, along with previously published cultivated eggplants (‘Gui’ and ‘SME’), three congeners (potato, pepper and tomato), an outgroup Taxus chinensis, and additional species. We identified 144 766 gene families, with ‘Y11’ and ‘Gui5’ containing 20 356 and 21 747 gene families, respectively, averaging 1.8 genes per family—similar to other Solanaceae members (Table S9). Analysis of shared and unique gene families revealed 3993 core gene families common to all 27 species (Fig. 2B). ‘Y11’ and ‘Gui5’ exhibited 2270 and 1328 unique gene families, respectively. Enrichment analyses highlighted both commonalities and distinct features in adaptive responses, defense mechanisms, and metabolic pathways between the two varieties. ‘Gui5’ exhibited notable gene enrichment in pathways related to plant–pathogen interactions, plant hormone signal transduction, and specific secondary metabolic pathways such as alkaloid biosynthesis (Fig. S3). At the same time, it was resistant to bacterial wilt (Table S22). This suggests a high level of adaptability to environmental stressors and robust defense mechanisms against pathogens. Conversely, ‘Y11’ showed enriched genes in plant–pathogen interactions, plant hormone signaling, flavonoid biosynthesis, phenylpropanoid biosynthesis, and photosynthesis pathways (Fig. S4). It has also been proven to have resistance to bacterial wilt (Table S22). Compared with *S. melongena*, *S*. *aethiopicum* has been reported to exhibit markedly lower susceptibility to pests and diseases and demonstrates a higher level of drought- and heat-tolerance under field conditions. Additionally, *S. aethiopicum* was used as a source of the disease resistance gene in breeding and could be crossed with cultivated eggplants to improve bacterial wilt resistance, making it a valuable rootstock for tomato [5, 6]. Based on the above research, it can be judged that ‘Y11’ possesses strong abilities to withstand external pressures while also enhancing secondary metabolite production and achieving efficient photosynthesis.

To further investigate the functional characteristics of these two eggplant varieties, we constructed a phylogenetic tree encompassing 27 species (Fig. 2A). Utilizing this framework, we conducted KEGG enrichment analysis of the expanded gene families in both ‘Gui5’ and ‘Y11’. This analysis unveiled their distinctive traits related to adaptability, defense mechanisms, and metabolic efficiency. ‘Gui5’ demonstrated significant gene expansion in plant–pathogen interaction and hormone signaling pathways, highlighting its adaptive capacity for enhancing disease resistance and growth regulation (Fig. S5). In contrast, ‘Y11’s gene expansion was significantly enriched in pathways related to photosynthesis antenna proteins, carbon fixation, as well as carbohydrate and fatty acid metabolism routes—reflecting its biological advantages in improving photosynthetic efficiency and energy conversion (Fig. S6).

WGD analysis across Solanum species, in comparison with grape, revealed that all Solanum species have experienced two distinct WGD events. These events are estimated at *K*s = 0.75 (approximately 53.57 million years ago, mya) and *K*s = 1.5 (approximately 107.14 mya). Notably, the *K*s = 1.5 event corresponds to the ancient γ-WGD event, which was also experienced by grape (Fig. S7). In this study, we delved into the ancestral chromosomal-level genome evolution within the Solanaceae family, centering our investigation on seven representative species: four variants of *solanums* (‘Gui5’, ‘Gui’, ‘SME’, and ‘Y11’), along with *S. tuberosum*, *S. lycopersicum*, and *C. annuum*. Our analysis revealed that chromosomes within the Solanaceae genus exhibit a high degree of relatedness, having undergone similar chromosomal breakage and fusion events. Nonetheless, certain chromosomal regions manifest considerable divergence. Notably, in the African eggplant compared to its three cultivated counterparts, there is an increased frequency of breakage and fusion in the mid-anterior segment of chromosome 1, alongside substantial deletions at the onset of chromosome 2 (Fig. 2C). These revelations were further substantiated by synteny analyses. In examining the syntenic and genetic structural relationships between the African eggplant ‘Y11’ and cultivated varieties ‘Gui5’, as well as previously documented ‘Gui’ and ‘SME’ genomes, we discerned superior collinearity among the three cultivated strains. This suggests that these cultivated varieties maintain a high degree of genetic structural consistency (Fig. 1D). Conversely, interchromosomal collinearity with the wild relative ‘Y11’ was less pronounced, marked by more significant genomic SVs. This suggests that throughout its evolutionary trajectory, the wild relative may have been subjected to distinct selective pressures or adaptive changes. Such findings illustrate that cultivated varieties are subject to more consistent human-driven selection and domestication trends while the wild variant embodies greater genetic diversity. Average Nucleotide Identity (ANI) calculations among Solanaceae species revealed that ‘Y11’ shares only 98.7% similarity with other eggplant cultivars while similarity among the other three cultivars exceeds 99.6% (Fig. 2A). This marked difference not only reflects greater retention of genetic diversity within wild ‘Y11’ but also elucidates its degree of genetic separation from cultivated types.

### Construction and analysis of the eggplant pan-genome

This research undertook a comprehensive construction of the eggplant pan-genome through both noniterative and iterative methods, integrating genomic data from the African eggplant ‘Y11’ and three cultivated varieties—‘Gui5’, ‘Gui’, and ‘SME’. The initial version (V1) used ‘Gui5’ as a reference framework, enabling the identification of extensive SVs through alignments with ‘Y11’, ‘Gui’, and ‘SME’. These included significant deletion variants, predominantly from ‘Y11’, contributing 1440 deletions totaling 138.95 Mb—key sources for additional sequences in the pan-genome (Tables S10 and S11). Subsequently, iterative assembly using whole-genome sequencing (WGS) data from 22 representative samples enriched the pan-genome further (Tables S12 and S13). The finalized eggplant pan-genome encompasses approximately 1.58 Gb and comprises 84 599 predicted protein-coding genes. Additional sequences derived from three high-quality assembled genomes have contributed 99.46 Mb, while iterative assembly from the resequencing of 22 samples has added an additional 337.48 Mb, culminating in a nonreference region (NRR) totaling 463.94 Mb (Table S14). These NRR sequences constitute 27.65% of the entire pan-genome size and include 44 299 genes, of which only a mere 6.29% (encompassing 4727 genes) originate from the three high-quality assembled genomes (refer to Tables S15 and S16). Through the construction of this pan-genome, we have substantially broadened our understanding of eggplant genetic diversity and illuminated the intricate variations and genetic resources within the eggplant genome. These achievements provide invaluable resources and a foundational basis for further research into eggplant gene function and genetic improvement.

**Figure 3:**
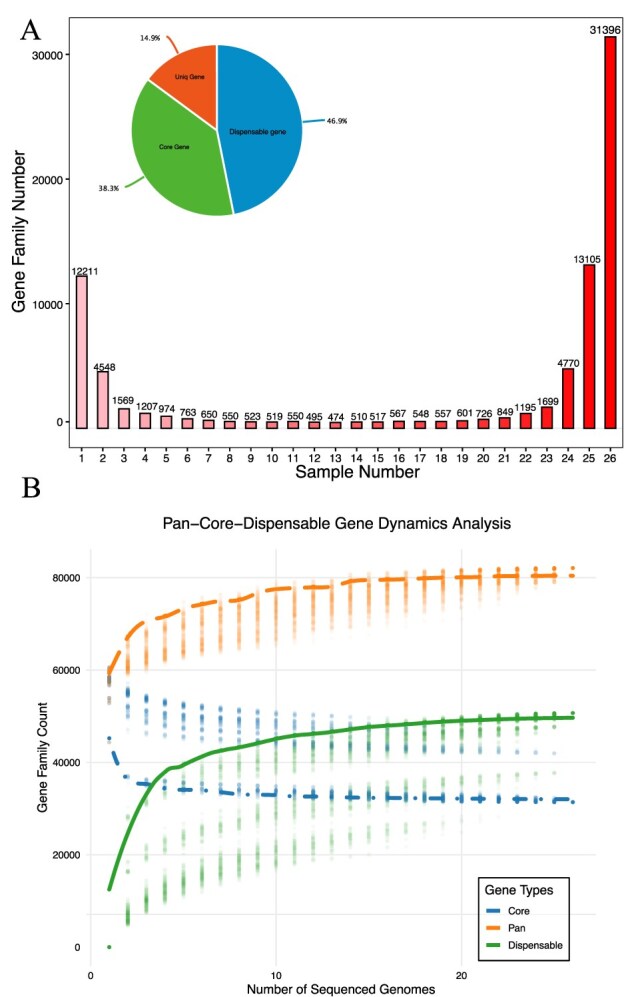
Pan-genome construction and core genome analysis: shared gene family statistics and gene count dynamics. **A.** This figure provides a statistical overview of the number of gene families shared across varying numbers of species. The pie chart in the top left corner illustrates the proportions of core genes, dispensable genes, and unique genes. **B.** The line graph depicts the dynamics of core and noncore genes. The stabilization and saturation of these curves suggest that the pan-genome is relatively comprehensive, indicating substantial coverage of the group.

This study’s analysis of the eggplant pan-genome and core genome elucidated its genetic diversity and dynamics. The growth curve of the pan-genome reached saturation beyond a specific sample threshold, indicating stability at 99% inclusion of total gene content within eggplants—demonstrating characteristics of a closed pan-genome system (Fig. 3B). Conversely, the core genome diminished in size with each addition of new genetic types, reflecting essential genes shared across all varieties. KEGG enrichment analysis highlighted that core genes are predominantly involved in metabolic pathways critical for growth and adaptation. The results ultimately reveal that core genes—defined as the set of genes consistently found across all individuals—comprise a mere 38.3% of the total genetic composition. In contrast, dispensable genes, which are present in some but not all individuals or strains within the group, constitute 46.9%, while unique genes represent 14.9%. (Fig. 3A). Through enrichment analysis of genes associated with presence–absence variations (PAV, which refers to SVs that are present or absent in specific cultivars), we discovered that these genes are enriched in pathways associated with metabolic regulation (such as glutathione metabolism, fatty acid metabolism, and amino acid biosynthesis), signal transduction (including calcium signaling pathway, mTOR signaling pathway, and plant hormone signal transduction), stress resistance (like plant–pathogen interaction and antioxidant-related pathways such as flavonoid biosynthesis), as well as growth and development (for example, cell cycle, regulation of actin cytoskeleton, and hormone-related pathways like auxin and cytokinin signal transduction) (refer to the attached data for details). These findings elucidate the potential roles these genes play in enabling eggplant adaptation to specific environmental conditions. Not only do these discoveries enhance our comprehension of the structural and functional intricacies of the eggplant genome, but they also provide a crucial molecular foundation for future genetic improvement targeting specific traits.

Pan-genomic analysis divided the materials into five groups according to geographical sources: Africa (AFR, wild species), Southeast Asia (SEA, primary cultivated species), Europe (EUR, advanced cultivated species), the southern China (SOU, advanced cultivated species), and the northern China (NOR, advanced cultivated species). The median numbers of core, dispensable, and unique genes were quantified for each group (Table S23). Core genes remained identical across all five groups (median = 31 396), consistent with the idea that core genes are evolutionarily conserved. While there were significant differences in the number of unique genes among wild species in Africa (1.362), advanced cultivated species in the northern China (1536), and significantly lower in southern China, Southeast Asia and Europe (80 118 and 61). African wild species have rich species diversity and complex natural habitats, which may prompt them to accumulate a large number of specific genes. In the process of long-term artificial domestication, the cultivated species in the northern China also retained many specific genes because of the difference of ecological conditions and strong selection pressure. In contrast, the environment of cultivated species in Southeast Asia, Europe, and the southern China is relatively simple, the history of artificial intervention is short or the selection direction is concentrated, resulting in a small number of specific genes.

### Population genomic analysis

Through comprehensive re-sequencing of 238 eggplant germplasm accessions, this study provides an in-depth assessment of their genetic diversity. The germplasm collection comprises 162 Chinese cultivars, including 87 from northern regions and 75 from southern areas, along with 27 Southeast Asian varieties, 48 European varieties, and a singular material from Africa (Table S17). The sequencing achieved an average depth of 11.45x, with a range spanning from 7.64x to 16.35x (Table S17). Alignment to the constructed pan-genome yielded an average alignment rate of 96.16%, with a range from 89.53% to 99.49% (refer to Table S18). In total, the study identified 7 853 531 single nucleotide polymorphisms (SNPs), 819 206 short insertions and deletions (indels) less than 15 bp, and 8 672 737 SVs (Table S19 and Fig. S8). The primary genetic variations are concentrated across the twelve chromosomes; however, fewer variations are observed within the additional sequences of the pan-genome, particularly those from three other genomes and E50 (Fig. S8). Furthermore, regions enriched in genes at chromosomal termini exhibited greater variation compared to the more conserved repetitive sequences abundant in central regions.

**Figure 4:**
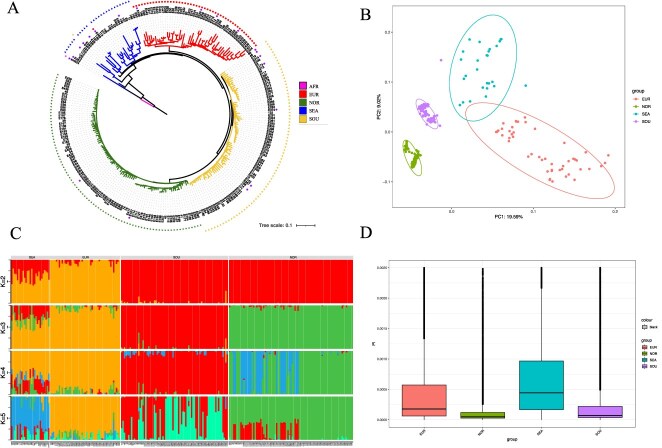
Population genomic analysis of eggplant. **A.** An unrooted phylogenetic tree constructed from core SNPs, encompassing 238 eggplant accessions. The accessions are categorized by their geographic origins: Africa, Southeast Asia, Europe, Northern China, and Southern China. For the purpose of pan-genome construction, selected varieties are marked with a distinctive star symbol accompanying their respective sample names. **B.** PCA plot showing the first two eigenvectors of the 238 accessions. **C.** ADMIXTURE analysis plot (*K* = 2–5) derived from core SNPs for the same set of accessions. **D.** Nucleotide diversity analysis across four regional varieties, with the vertical axis representing the PI value. A lower PI value suggests reduced nucleotide diversity at a specific locus within the population, implying stronger selection pressures, whether natural or otherwise. The PI values range between 0 and 1.

An unrooted tree constructed using core SNPs classified the 238 eggplant germplasms into four distinct groups (Fig. 4A), a classification corroborated by both overall structure analysis (*K* = 4) and principal component analysis (PCA) (Fig. 4B and C). This indicates these methodologies effectively capture the genetic similarities and disparities among the germplasms. The groupings align closely with geographical information, suggesting that regional factors significantly influence the genetic structure of eggplant germplasm. This likely reflects unique genetic traits shaped by geographic isolation, cultivation practices, or natural selection pressures across different regions. The evolutionary tree based on SNPs suggests that eggplant was domesticated in Southeast Asia earlier than in Europe and China, offering critical insights into their geographical dispersion and domestication history (Figs 4A and S9). Through a comprehensive analysis of gene flow, it has been discerned that three significant episodes of genetic exchange transpired among these four groups (Fig. S10). One such event occurred as gene flow from the southern China into the Southeast Asian region, marking the sole instance of gene flow within the group itself; the other two instances involved external populations. This mechanism aptly aligns with both the geographical distribution characteristics of eggplants and the patterns of genetic interchange. Analyses suggest that both European and Chinese eggplants were independently domesticated, occurring after the domestication of eggplants in Southeast Asia. Subsequently, further differentiation within China led to the emergence of distinct southern and northern lineages. This intriguing pattern reveals that upon their arrival in China, eggplants did not adhere to a conventional north-to-south or south-to-north propagation trajectory, each following separate routes.

The PI values from nucleotide diversity analysis indicate higher diversity among Southeast Asian and European varieties compared to relatively lower diversity among Chinese northern and southern varieties (Fig. 4D). This might imply that Southeast Asian and European eggplant germplasm underwent more complex historical genetic exchanges or retained more wild genetic resources. Conversely, the reduced diversity in Chinese varieties may reflect strong selection pressures during introduction and domestication processes, which diminished genetic variability. Comparative analyses of differentiation among different groups reveal significant divergence between Chinese northern/southern groups and Southeast Asian groups but minimal divergence between the northern and southern groups within China (Fig. S11). This suggests that Chinese northern/southern eggplant germplasm likely shares a similar origin from common ancestral stock and underwent rapid domestication and cultivation over a relatively short period. The larger differentiation from Southeast Asian groups may reflect genetic drift due to geographic isolation or different environmental selection pressures. In sum, these findings provide crucial genetic insights into the conservation and utilization of eggplant germplasm resources.

### SNP-based GWAS of agronomic traits

In this study, we collected phenotypic data for 10 agronomic traits from 235 eggplant specimens cultivated in Nanning, Guangxi, during the years 2020 and 2021. The analyses revealed a high level of consistency in these phenotypic characteristics across the different years. The ten agronomic traits examined include fruit surface grooves (FSG), single fruit weight (SFW), fruit longitudinal diameter (FLD), fruit transverse diameter (FTD), length-to-width ratio (LWR), circumference (CIR), number of chambers (NOC), thousand seed weight (TSW), number of seeds per fruit (NSF), and fruit navel shape (FNS) (Table S20). By integrating the mean phenotypic data over two years for a genome-wide association study (GWAS), we identified 946 significant SNP-trait associations (STAs) (Table S21). Although most STAs were located in intergenic regions, 9605 related genes were identified. Notably, some STAs were shared among highly correlated traits, with 2053 genes appearing in association with more than two traits and 886 genes involved in three or more associated traits (Fig. S12).

Further analysis revealed that certain genomic regions appeared to be hotspots for STAs, particularly those associated with yield-related traits. We discovered that 157 genes were associated with more than four traits, and only three genes were concurrently associated with five traits. These three contiguous genes—*sm005_gene028240.1*, *sm005_gene028241.1*, and *sm005_gene028242.1*—are associated with SFW, FTD, FLD, FSG, and CIR, all of which are key agronomic traits closely linked to eggplant yield. Interestingly, these loci reside within additional sequences constructed from the pan-genome rather than the original chromosomal genome. This underscores the limitations of using a single species as a reference genome and highlights the significance of the pan-genome for GWAS analyses. The pan-genome not only supplements the diversity sequences of species populations more comprehensively but also prevents the omission of specific sequences or loci. Although the functions of these three genes remain unclear, functional annotation suggests that *sm005_gene028241.1* may be linked to transposable elements, indicating its role in DNA movement and recombination within the genome. These regions may be closely associated with domestication selection in eggplants.

**Figure 5:**
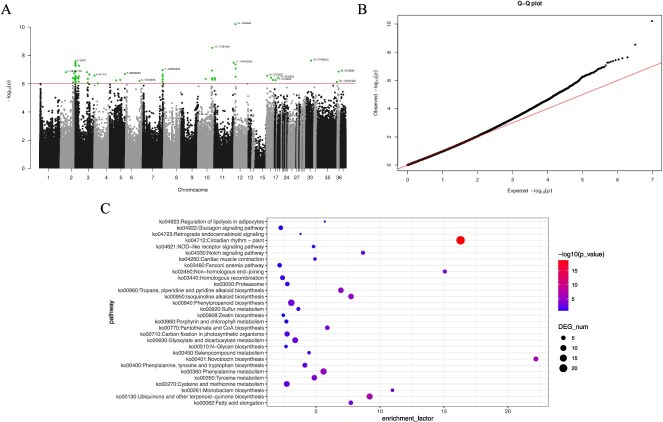
Genetic association study and functional enrichment analysis of ribbing traits on eggplant fruit surface. **A.** Manhattan plot of the genome-wide association study (GWAS) for ribbing traits on the surface of eggplant fruit. Data points above the significance threshold line indicate loci significantly associated with ribbing traits, with some of the loci already annotated. **B.** Q–Q plot of GWAS results for ribbing traits, illustrating the observed versus expected distribution of *P*-values. The plot shows the deviation of observed *P*-values from the null hypothesis, indicating potential associations. **C.** KEGG pathway enrichment analysis for genes associated with ribbing traits on the fruit surface. Enriched pathways highlight biological processes potentially involved in the development of ribbing traits.

Fruit surface grooves (FSG) represent a critical phenotypic trait of eggplant fruit appearance. Analysis through the Manhattan and Q–Q plots demonstrates a strong association of GWAS-identified loci with this trait, with significantly associated genes enriched in several key biological pathways (Fig. 5). This reveals the complex regulatory mechanisms underlying its formation. Firstly, the enrichment in the Circadian rhythm—plant and Notch signaling pathways suggests that plant circadian rhythms and cell differentiation play crucial roles in FSG formation. Circadian rhythms may influence the growth patterns of the fruit surface by regulating cell cycle and metabolic rhythms, while the Notch signaling pathway could affect fruit appearance by modulating cell fate and tissue patterning. Furthermore, the enrichment of pathways such as Proteasome and Homologous recombination implies potential roles for protein degradation and DNA repair mechanisms in shaping this phenotype. The proteasome is involved in protein degradation and cell cycle regulation, possibly influencing fruit surface structure by affecting cell growth and differentiation. Meanwhile, the enrichment of secondary metabolic pathways like zeatin biosynthesis and phenylpropanoid biosynthesis highlights the significance of plant hormones and secondary metabolites in FSG formation. Zeatin may impact fruit surface morphology by promoting cell division and expansion, whereas phenylpropanoids could regulate fruit appearance and texture by affecting cell wall composition and mechanical properties. The synergistic effects of these pathways reflect a multilayered biological regulatory network involved in the formation of FSG, showcasing their intricate genetic and biochemical underpinnings.

Gene enrichment analysis on significantly associated genes for the ten agronomic traits revealed multiple phenotypes significantly enriched in common biological pathways such as the GnRH signaling pathway, zeatin biosynthesis, biosynthesis of amino acids, sulfur metabolism, and phenylalanine, tyrosine, and tryptophan biosynthesis/metabolism (Figs S13–S21). These pathways primarily involve plant hormone signaling transduction as well as biosynthetic and metabolic processes, suggesting their potential roles in eggplant growth, development, and environmental adaptation. Particularly noteworthy is zeatin biosynthesis due to its close relationship with plant auxins; it may significantly affect fruit size and shape attributes like circumference and longitudinal diameter. SFW and TSW are especially critical among phenotypes influencing eggplant yield. Relevant pathways such as mTOR signaling pathway and protein processing in endoplasmic reticulum play pivotal roles in cell growth regulation and protein-folding quality control, respectively; these may modulate fruit size by affecting cell proliferation, protein synthesis, and cellular function. Additionally, pathways like amino acid biosynthesis and fatty acid metabolism provide essential foundations for protein synthesis and energy storage supporting fruit growth and development. Pathways that warrant particular attention include the circadian rhythm—plant and Notch signaling pathway, which regulate diurnal rhythms in plants as well as cell differentiation and fate determination—key processes influencing fruit structure, shape, and growth patterns. Meanwhile, the enrichment of the mTOR signaling pathway across multiple phenotypes highlights its potential role in regulating growth rate, resource allocation, and developmental regulation. Investigating these pathways is of significant scientific value for elucidating genetic mechanisms, guiding targeted genetic improvement, and enhancing crop yield and quality. Moreover, it offers comprehensive insights into the coordination of genetic and biochemical networks. The unique overlap among these pathways underscores the complexity of phenotype expression, which is driven by the integration of metabolic, hormonal, and environmental signals. These findings have promising applications for agricultural improvement and food security.

## Discussion

High-quality T2T chromosome-level genomes are crucial for crop genetics and breeding. In this study, we successfully assembled two T2T-level eggplant genomes—the African eggplant ‘Y11’ and the cultivated eggplant ‘Gui5’—by integrating high-depth HiFi sequencing ONT Ultra-Long sequencing and Hi-C sequencing technologies. We predicted 37 324 and 40 300 protein-coding genes for each genome, respectively, using evidence from *de novo* prediction, homologous proteins, and RNA-seq. Compared to the previously published cultivated eggplant genomes Gui [17] and SME [16], our genomic assemblies exhibit significant improvements in quality, with contig N50 increasing from approximately 5 Mb to 94.2 and 93.9 Mb, respectively (Table 1). During gene annotation, we noted areas for improvement in some gene models. Although most gene models are supported by RNA-seq or homology evidence, some protein-coding genes appear short, potentially being partial, fragmented, redundant, or chimeric, necessitating further experimental validation and manual curation. Despite these issues, the total gene numbers are comparable to those of existing eggplant genomes, with excellent protein collinearity indicating minimal impact on overall genomic integrity. Potential false positives, such as small open reading frames lacking homologous evidence, have a negligible effect on our conclusions drawn from functional enrichment analyses. Additionally, we selected 22 representative samples based on regional and phenotypic representation for deep resequencing. Alongside the published ‘Gui’ and ‘SME’ varieties, these contributed to constructing an eggplant pan-genome that enhances our understanding of the entire genome space and identifies genes associated with key traits through SNP-based and PAV-based GWAS.

Although the first pan-genome for eggplants was published in 2021 [15], it relied heavily on public database resources without significant overlap with our study’s varietal resources or regional origins. It lacked representativeness of Chinese and Asian varieties due to scarcity in authentic phenotypic information and resources. Our newly released pan-genome not only includes four high-quality chromosome-level reference genomes but also possesses regional representativeness characteristic of China and Southeast Asia. This framework is more comprehensive with richer additional pangenomic sequences exceeding 430 Mb (Table S14), doubling the number of coding genes (Table S15), making it the highest quality dataset currently available among eggplant genomic resources. Through deep resequencing of 238 accessions from China, Southeast Asia, Europe and Africa, we conducted an in-depth evaluation of genetic variation. The SNP-based data derived from the pan-genome enabled us to classify Chinese eggplants into two independent groups that were highly consistent with the geographical distribution of north and south. Phylogenetic and population structure analyses revealed that domestication of eggplants in Southeast Asia predated that in Europe and China, and materials from this region showed higher nucleotide diversity and earlier differentiation time, supporting its status as the primary domestication center and center of genetic diversity. European and Chinese eggplants subsequently underwent independent domestication events following the initial domestication in Southeast Asia. These groups showed clear genetic clustering and limited gene flow between populations, with particularly significant genetic differentiation between northern and southern varieties in China, along with low genetic diversity, indicating long-term isolation and local adaptation. Chinese eggplants were likely introduced via multiple routes and underwent secondary diversification rather than a simple south-to-north migration. These findings not only corroborated the hypothesis of eggplant proliferation ‘Out of Africa, into the Orient’ but also aligned with the proposed the existence of at least two main centers of domestication in Southeast Asia and the Indian subcontinent [2,40,41]. Phylogenetic trees, structure plots, and PCA analyses further revealed unique genetic components and admixture patterns within Chinese populations, strengthening the inference of regional adaptation during domestication. Thus, this study provided robust genomic evidence for the complex domestication and migration history of eggplants, demonstrating that they underwent multiple independent domestication events followed by regional adaptive differentiation.

This study identified numerous genomic regions associated with agronomic traits, particularly highlighting the abundance of genes derived from the pan-genome—regions previously unattainable using a single genome. Notably, a region associated with five phenotypes involves three genes, all contributed by the additional sequences from the E469 material to the pan-genome. This underscores the substantial application value of the pan-genome in population genetics, revealing regions that were inaccessible with a singular genome approach, while also illustrating the limitations inherent in relying solely on a single genome. Furthermore, from the PAV genome, we uncovered several critical functional genes. For instance, we identified numerous pathways related to stress resistance, such as plant–pathogen interaction and antioxidant-related pathways like flavonoid biosynthesis. These essential PAV genes serve as crucial genetic resources for varietal breeding. The PAV genes specific to certain varieties are pivotal for deciphering eggplant’s genetic diversity and represent vital germplasm resources. In summary, the high-quality eggplant genome constructed in this study, along with genetic variation mapping and the pan-genome, lays a robust foundation for gene discovery and breeding in eggplant.

## Conclusion

In summary, the high-quality T2T reference genomes, the Asian-representative pan-genome, and the insights into population genetics and trait-associated genes generated in this study collectively provide a comprehensive genomic resource. These resources will not only deepen our understanding of eggplant evolution, domestication, and trait formation but also serve as powerful tools to accelerate the development of high-yield, stress-tolerant, and high-quality eggplant varieties, driving significant progress in global eggplant breeding.

## Materials and methods

### Plant materials, library construction, and sequencing

For whole-genome sequencing, the African eggplant ‘Y11’ and the cultivated eggplant ‘Gui5’ were selected. ‘Y11’, a wild relative of eggplant (*S*. *aethiopicum*), was collected from Xiadong Town, Longzhou County, Chongzuo City, Guangxi Zhuang Autonomous Region, China. It thrives optimally at temperatures between 25°C and 30°C and produces flattened round fruits with shallow green skin when semi-ripe and deep red skin with prominent ridges when fully ripe. The cultivated variety ‘Gui5’ is a disease-resistant, high-yielding, high-quality stable line developed through six generations of self-crossing by the Vegetable Institute of Guangxi Academy of Agricultural Sciences. It is characterized by its long, purple-red fruit.

DNA was extracted using the CTAB method from leaves of both varieties and then fragmented via sonication for genomic sequencing [42]. To ensure high-quality genomic data, libraries with 350 bp insert sizes as well as 15 kb SMRTbell libraries were constructed. Sequencing for long-read libraries was performed on the PacBio Revio platform to generate subreads. Consensus reads (HiFi reads) were derived using PacBio’s official SMRT Link software ccs module. For Illumina short-read sequencing, protocols provided by the manufacturer were followed on the Illumina NovaSeq 6000 platform.

For the library preparation methods employed in ONT ultra-long sequencing, one begins by extracting high-quality DNA samples, which are then subjected to rigorous quality control assessments. These assessments include evaluating color, purity, concentration, and integrity of the samples. Once this is accomplished, magnetic bead selection is utilized to isolate target fragments followed by processes for damage repair and end-repair. Subsequently, the DNA undergoes purification before sequencing adapters are ligated. The library is purified again and precisely quantified. Ultimately, the library is loaded onto an R10.4.1 Flow Cell and sequenced using a PromethION sequencer.

During Hi–C sequencing, nuclear DNA collected from young leaves was first crosslinked to capture interactions between DNA fragments [43]. The DNA was then digested using the restriction enzyme MboI (NEB, R0147M). Following biotinylation and ligation of these fragments, a sequencing library was constructed and sequenced on the Illumina NovaSeq 6000 platform.

### Genome assembly and evaluation

To assemble high-quality reference genomes for the two eggplant varieties, we utilized all HiFi reads as input for the hifiasm (v0.19.5) assembler [44], employing default parameters to generate contigs with excellent continuity. Subsequently, Hi-C reads were aligned with the draft assembly contigs, and chromosome scaffolding was performed using the 3d-DNA (v201008) software [45]. The juicer-box program was then employed to manually inspect and rectify misassemblies with abnormal Hi–C interaction matrices, resulting in the final set of 12 pseudo-chromosomes. Chromosome number and orientation were confirmed through whole-genome alignment with previously published genomes.

To address the gap regions within chromosome-mounted areas and achieve a complete T2T genome assembly, we initially employed Minimap2 to align raw Oxford Nanopore Technologies (ONT) data against preliminary chromosomal assemblies. Subsequently, Racon was utilized for gap closure. Upon completing the gap bridging, we applied the Pilon program by incorporating high-quality short-read data from NGS to refine and polish the assembly further. To resolve any lingering gaps, HiFiasm was deployed once more to perform hybrid assembly with both HiFi and ONT datasets, producing contigs that were then aligned using BLAST (v2.12.0) against the chromosomal genome for manual gap filling. This iterative process was repeated as needed until all gaps were meticulously addressed and closed.

To assess the completeness of the eggplant genomes, we first examined the presence of telomeric motifs—5′ end CCCTAAA or 3′ end TTTAGGG—on each assembled chromosome. We also employed quartet (v1.1.6) tool to identify candidate centromeric sequences [46].

For evaluating genome accuracy, we implemented three complementary methods: BUSCO analysis [47], HiFi read alignment, and LTR Assembly Index (LAI) assessment. BUSCO analysis was conducted using BUSCO_V4 based on the embryophyta_odb10 database for land plants. HiFi reads were aligned to their respective reference genomes using Minimap2 [48], achieving an alignment rate of 99.86% for both ‘Gui5’ and ‘Y11’. To ascertain the LAI [49], we employed annotated LTR sequences. The resulting LAI values were 16.66 for ‘Gui5’ and 14.44 for ‘Y11’, classifying these genomes as Gold-standard quality. Finally, assembly completeness was further evaluated using the Merqury software, which computed consensus quality values (QV). The QV for ‘Gui5’ was determined to be 59.24, while ‘Y11’ exhibited a QV of 58.57, providing insight into the relative assembly accuracy and integrity of each genome.

### Annotation

In order to identify repeat contents in two eggplants, we combined *de novo* prediction with homology-based prediction methods [50]. Initially, a *de novo* repeat library for each eggplant was constructed using RepeatModeler (v2.0.1; http://www.repeatmasker.org/RepeatModeler/) [51]. RepeatModeler automatically invoked TRF (v4.09) [52], RECON (v1.08) [53], and RepeatScout (v1.0.6) software to construct models of repetitive sequences, and employed the MISA (v1.0) script tool for microsatellite sequence (SSR) prediction [54]. Subsequently, LTR identification was performed separately using LTR_finder (v1.0.7) [55] and LTR_harvest (v1.5.11) [56], followed by integration with LTR_retriever (v2.7) to obtain complete LTR sequences [56]. Furthermore, multiple *de novo* repeat libraries from each genome were merged using Repbase, and the merged libraries were input into RepeatMasker (v4.1.0; http://www.repeatmasker.org/RepeatMasker/) to annotate the repetitive elements of the assembled genomes [57].

We employed the same annotation pipeline for the genomes of two eggplants, utilizing three distinct methodologies to predict protein-coding genes within their genomes. These approaches encompassed *de novo* gene prediction, homology-based gene prediction, and RNA-Seq evidence-based gene prediction [58]. Prior to gene prediction, we applied RepeatMasker to mask repetitive sequences within the genomes of both eggplants. Initially, *de novo* gene prediction was performed using the Augustus tool (v3.3.3) [59], with each model constructed based on high-quality protein sequences generated from RNA-Seq datasets. Subsequently, homology-based gene prediction was executed by aligning protein sequences and transcript sequences from closely related species to the reference genome, in conjunction with maker (v2.31.10) [60]. As for RNA-Seq evidence-based gene prediction, preprocessed RNA-Seq reads were aligned to the genome using hisat2 (v2.0.0) initially [61]. This was followed by integration of Trinity (v2.3.2) [62], Transdecoder (v2.01) [63], and maker in combination, facilitated by the ‘—genome_guided_bam’ parameter, to accomplish predictions of protein-coding gene structures. Ultimately, leveraging EVidenceModeler (EVM, v1.1.1) [64], we integrated the predictions generated by these three methods into a nonredundant set of gene structures for final output.

In order to elucidate the functionality of predicted protein-coding genes, we consulted the esteemed repositories of biological information, including the National Center for Biotechnology Information (NCBI) Nonredundant (NR), TrEMBL, KOG, and Swiss-Prot protein databases [65], as well as the Kyoto Encyclopedia of Genes and Genomes (KEGG) database [66]. Initially, an alignment analysis was conducted using the BLASTP tool (ncbi blast v2.6.0+) against these databases [67], employing an *E*-value threshold of 1E-5, in quest of optimal matches for protein functional annotation. Subsequently, the identification of protein domains was accomplished through PfamScan (v1.6) tool in conjunction with PFAM and InterPro protein databases for annotation purposes [68]. The Gene Ontology (GO) IDs for each gene were obtained using the Blast2GO software suite [69].

### Evolutionary analysis

In this investigation, we employed an array of bioinformatics techniques for evolutionary analysis to explore the clustering of gene families and the phylogenetic history across 27 diverse species. Initially, using OrthoFinder (v2.5.5) [70], we conducted a diamond blastp alignment of all protein sequences to discern homologous gene pairs (*E*-value <1*e*-5, minimum coverage >40%). Subsequently, these homologous gene pairs were clustered into families using the mcl algorithm, thus organizing information on gene families.

To construct a phylogenetic tree for the 27 species under study, our research retrieved 1614 conserved single-copy genes from the BUSCO embryophyta_odb10 database. The longest isoforms of these genes in each species were selected for analysis. We aligned their coding sequences (CDS) globally using the muscle program and subsequently utilized raxml software to build the evolutionary tree [71]. Integrating fossil calibration data from the timetree database [72], we estimated speciation times using an mcmc method [73], ultimately presenting a temporally annotated phylogenetic tree. Furthermore, by inputting both the phylogenetic tree and gene family clustering results into the cafe program [74], we analyzed significant events of gene family contraction and expansion (*P* < 0.05) [75].

To assess WGD events within Solanaceae species, data from Vitaceae was incorporated. We compared intraspecies protein sequences via blast (*E*-value <1*e*-5, minimum coverage >40%), and employed MCScanX (v1.0.0) to identify syntenic blocks containing homologous genes [76]. Protein sequences and CDSs of these homologous genes were then subjected to alignment with ParaAT (v2.0) (utilizing MAFFT v7.471) [77], followed by *K*a/*K*s ratio analysis employing KaKs_Calculator to derive *K*s frequency distribution graphs [78]. Additionally, JCVI (v1.3.6) software was employed to generate synteny maps for Solanaceae species [79]; by aligning with previously published models of ancestral eudicot chromosomes (AEK), we discerned ancestral chromosomal origins within each genome and presented visual depictions thereof. This comprehensive suite of analyses elucidated genetic diversity and evolutionary dynamics in Solanaceae species, providing profound insights into their evolutionary lineage.

### Pan-genome construction

In the endeavor to construct the pan-genome of eggplant, this study employed a series of sophisticated bioinformatics steps. Initially selecting ‘Gui5’ as the reference genome, we aligned the genomes of Gui, SME, and Y11 against this scaffold. Utilizing the BWA (v0.7.17) program [80], we identified and integrated insertions exceeding 5 kb that lacked kmer reads coverage on the scaffold genome, thereby assembling version V1 of the species’ pan-genome. Furthermore, each genome’s integrated sequence was annotated for gene locations using the maker program to ensure accuracy and completeness.

To further refine the pan-genome, our study compiled 22 representative samples of eggplant materials and subjected them to NGS at a depth exceeding 30X. The resequencing data, which were processed following the iterative assembly approach published in *Nature Communications* [81], were utilized to construct the pan-genome. Initially, these data aligned to the V1 version of the pan-genome using bowtie2 (v2.5.1) [82]. Sequences that failed to align were extracted with samtools (v1.19) [83] and assembled *de novo* using spades. The resulting sequences underwent redundancy reduction with redundans program, filtered by criteria including similarity greater than 90%, coverage over 80%, and length exceeding 1000 bp. Subsequently, gene prediction on these redundancy-reduced assembled sequences was performed using augustus (employing a gene model suited for this species). Through these meticulous processes, we obtained fasta formatted assembled sequences and gff formatted annotation files.

Ultimately, these datasets were integrated into V1 of the pan-genome to create version V2 based both on genomic data and resequencing data. This comprehensive approach not only enhanced the coverage and accuracy of our eggplant pan-genome but also provided deeper insights into its genetic diversity. This lays a solid foundation for future genetic research and breeding endeavors in eggplants.

### Resequencing and variant calling

DNA was extracted from 238 eggplant samples and sequenced on the MGI BGI T7 platform. The sequencing data in fastq format were aligned to the pan-genome reference using Bowtie2 with default parameters. Sorting and removal of PCR duplicates were performed using Samtools and Picard (v3.1.1) tools. Genetic variants, including single nucleotide polymorphisms (SNPs) and short insertions and deletions (<15 bp), were detected using the Genome Analysis Toolkit (GATK, v4.0) [84], followed by filtering with VCFtools [85]. The criteria for SNP and InDel filtering included: a quality score greater than 30, read coverage per site above 5, minor allele frequency greater than 0.05, and a missing rate per site below 0.7.

The identified genetic variants were annotated according to the gene models of the Pangenome v2 reference genome using SnpEff (v5.0e) [86]. Additionally, VCFtools (v0.1.17) was employed to determine the density of variants across each chromosome, utilizing a 1-Mb sliding window approach [87].

### Population genetic diversity and structure analysis

Population genetic structure analysis serves as a critical tool for elucidating the ancestral origins and composition of individuals. Using SNP data, we employed the ADMIXTURE software with a block relaxation algorithm to ascertain the population structure of the samples [88]. Additionally, PCA clustering was conducted using GCTA software [89], and the first two eigenvectors were plotted.

To construct a phylogenetic tree for all samples, we utilized RAxML (v1.2.1) software with SNP data based on the maximum likelihood method. The highly ancestral wild eggplant sample ‘SM624’, hailing from Southeast Asia, served as the outgroup to establish the root of the phylogenetic tree. The resultant tree was visualized using the Interactive Tree of Life (iTOL) platform (https://itol.embl.de/) [90].

The SPAGeDi (v1.5d) software can be employed to estimate the relative kinship between individual pairs within natural populations [91], resulting in a kinship clustering heatmap for the samples. The nucleotide diversity (π) of the population was assessed using VCFtools with a sliding window approach set at 100 kb. Concurrently, genetic differentiation between populations was evaluated by calculating Fst indices using VCFtools with a sliding window size of 3 kb. This provided insights into the degree of differentiation between the two groups.

### Phenotyping and GWAS

In the experimental cultivation of eggplant materials, we observed a total of ten agronomic traits over two consecutive years. In the first year, each trait was recorded only twice, while in the second year, they were documented once. Consequently, their mean values were employed for downstream analysis. Detailed information regarding each trait can be found in Table S20. This study conducted an association analysis based on SNP and InDel marker data using the EMMAX program [92]. During the analysis, population structure and kinship matrix were incorporated to adjust the model. The significance threshold for trait associations was set at a *P*-value less than 1*e*-6. Significant SNP loci within 100 kb upstream and downstream were considered candidate gene regions. Candidate genes were selected from these regions and subjected to functional annotation and KEGG enrichment analysis using seven functional databases: NR, KEGG, Swiss-Prot, TrEMBL, KOG, GO, and PFAM.

## Supplementary Material

Web_Material_uhaf248
