# Supplementary material for: Graph pan-genome advances genetic discoveries and the improvement of eggplant
Source: Hortic Res. 2025 Sep 19;13(1):uhaf248. doi: 10.1093/hr/uhaf248 (PMC12854085; doi:10.1093/hr/uhaf248)
Supplement: Web_Material_uhaf248 [file web_material_uhaf248.zip › Supplementary Figures_Revised.pdf]

Supplementary Figures

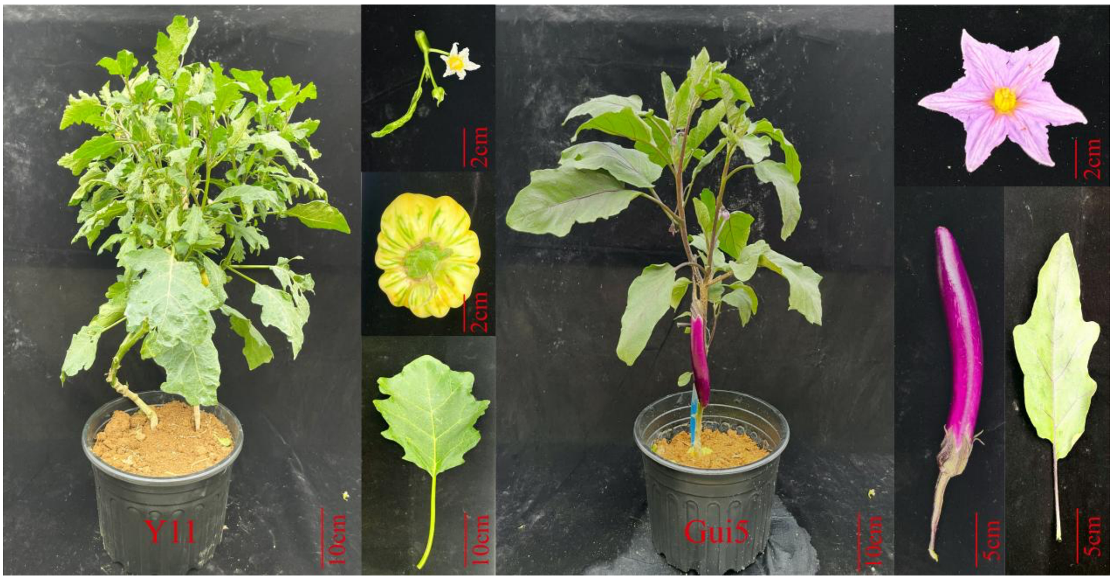

Figure S1. Whole plant, flower, fruit, and leaf phenotypes of Y11 and Gui5.

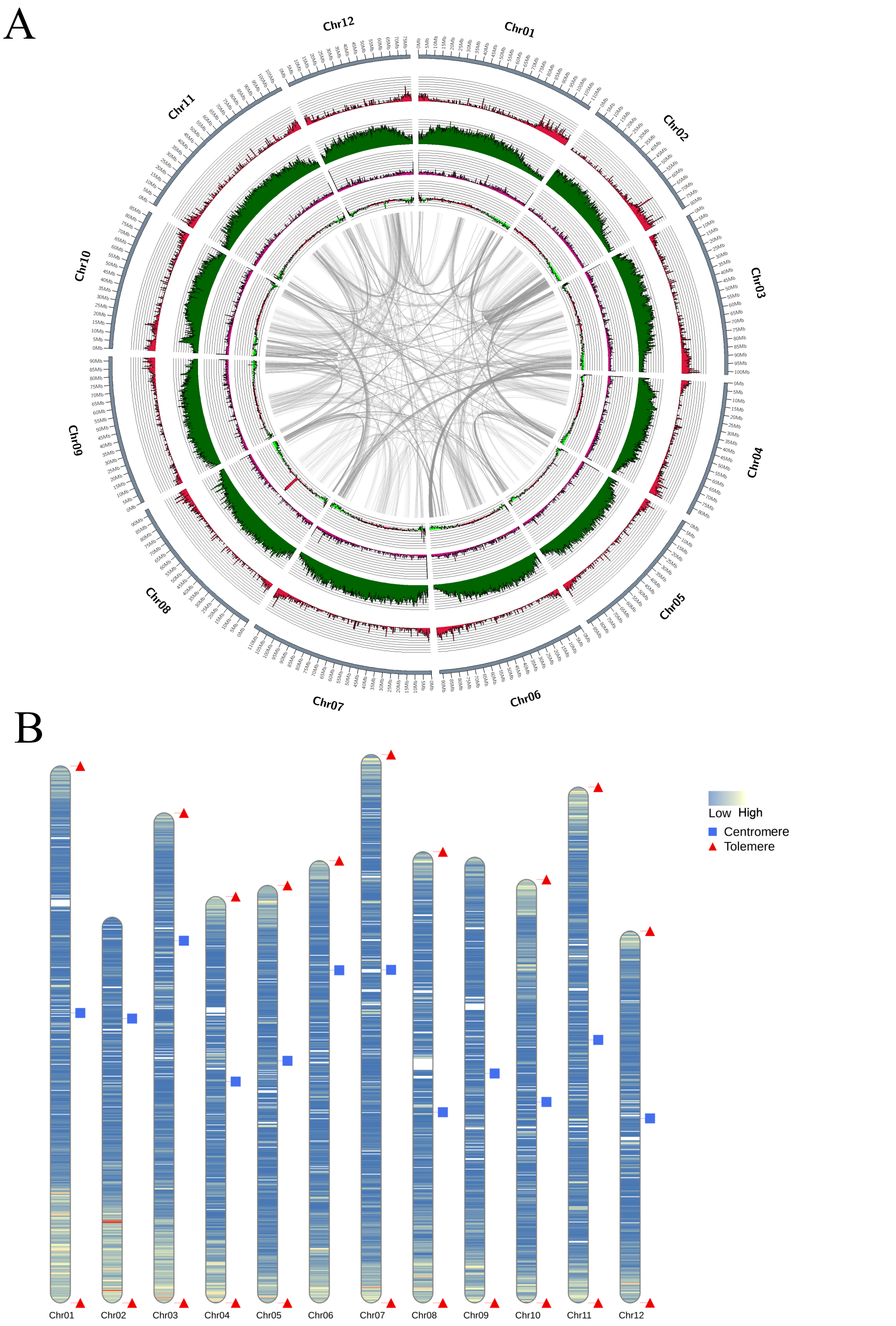

Figure S2. Overview of the Gui5 Eggplant Genome. A. The landscape of genomic features for Gui5 is depicted as a series of concentric circles, arranged from the outermost to the innermost: 1) gene density (200 kb sliding window); 2) transposon density (200 kb sliding window); 3) repeat sequence density; 4) GC content (200 kb sliding window, with values above the genome-wide average distinguished from those below average); 5) gene synteny blocks. **B.** Additionally, tel omere and centromere detection is illustrated. A gradient scale represents gene density, and number s indicate chromosome identifiers.

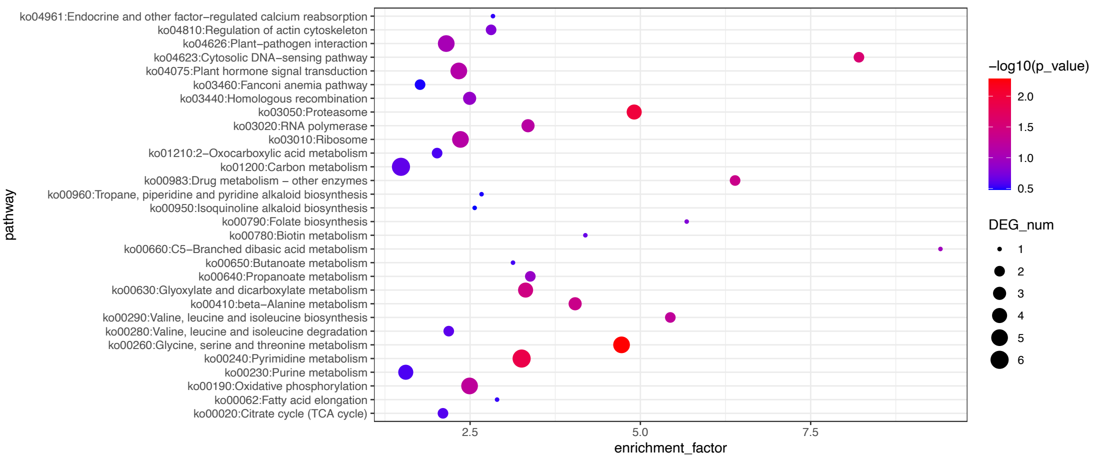

Figure S3. KEGG Enrichment Bubble Chart of Eggplant Gui5-Specific Genes in Comparative Evolutionary Analysis with 26 Other Species.

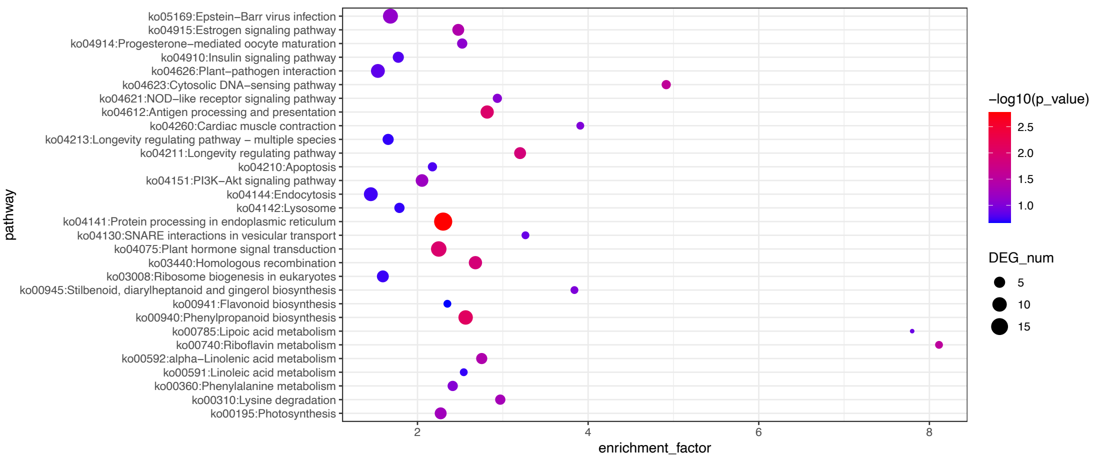

Figure S4. KEGG Enrichment Bubble Chart of Eggplant Y11-Specific Genes in Comparative Evolutionary Analysis with 26 Other Species.

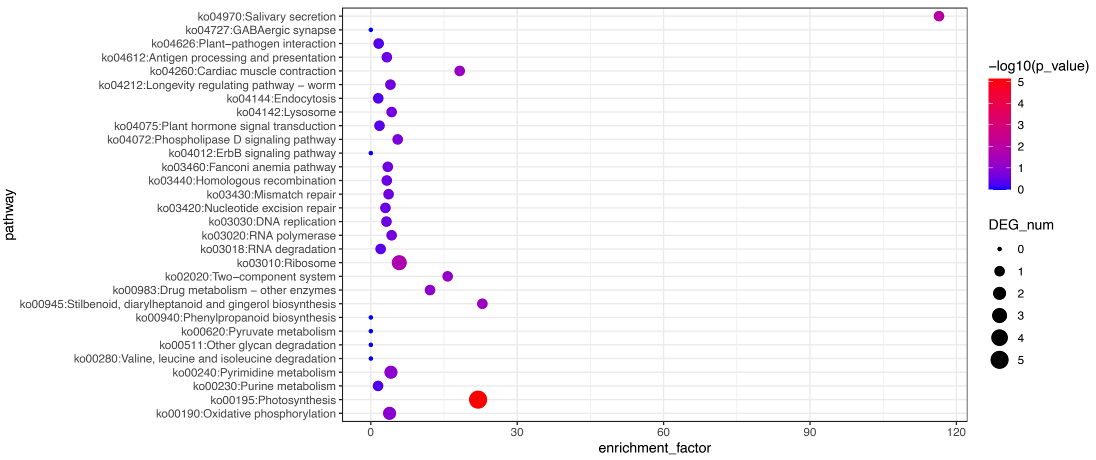

Figure S5. KEGG Enrichment Bubble Chart of Ancestrally Expanded Genes in Eggplant Gui5 from Comparative Evolutionary Analysis.

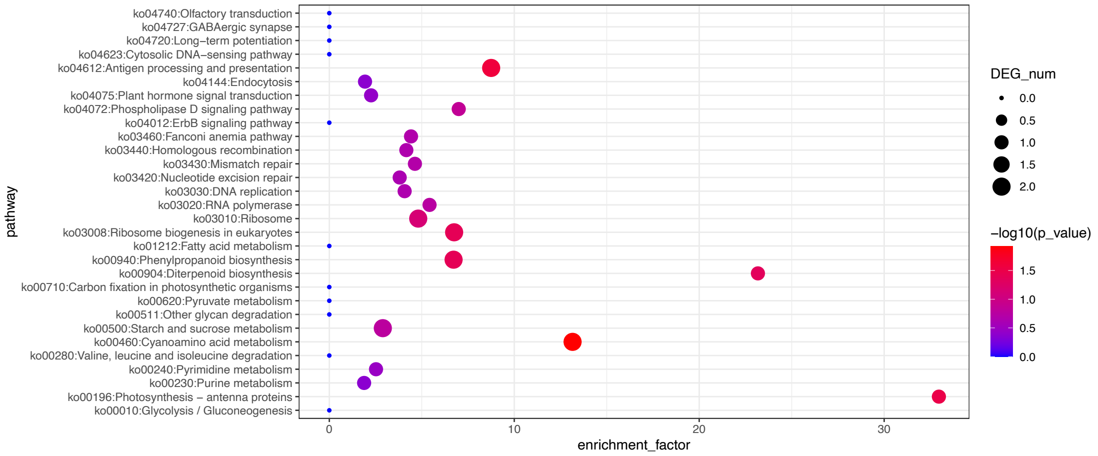

Figure S6. KEGG Enrichment Bubble Chart of Ancestrally Expanded Genes in Eggplant Y11 from Comparative Evolutionary Analysis.

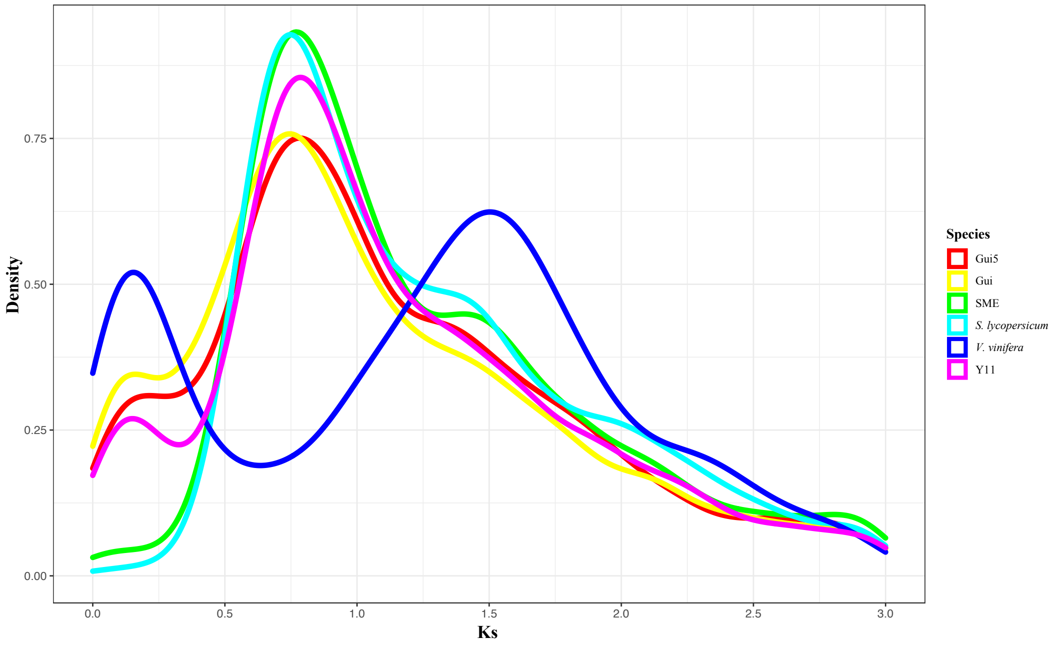

Figure S7. Ks Frequency Distribution of WGD Protein-Coding Genes Across Six Species (*V. vinifera*, *S. lycopersicum*, *S. melongena* Y11, SME, Gui and Gui5)

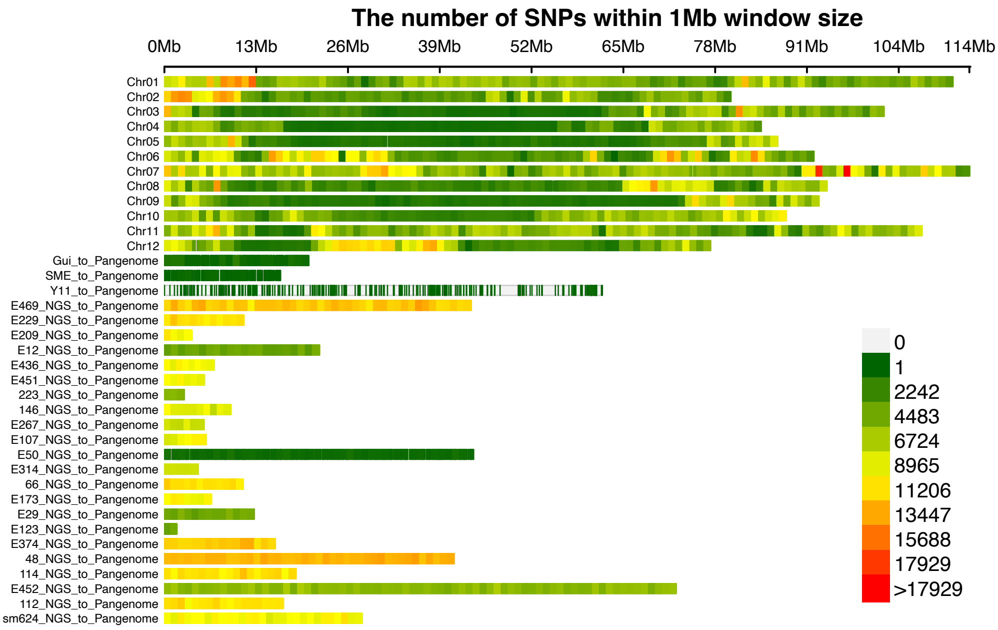

Figure S8. The chromosomal distribution of SNP variant loci. Aside from the 12 chromosomes, the contig sequences are derived from additional sequences shared among different samples. The figure presents SNP density with a window size of 1 MB.

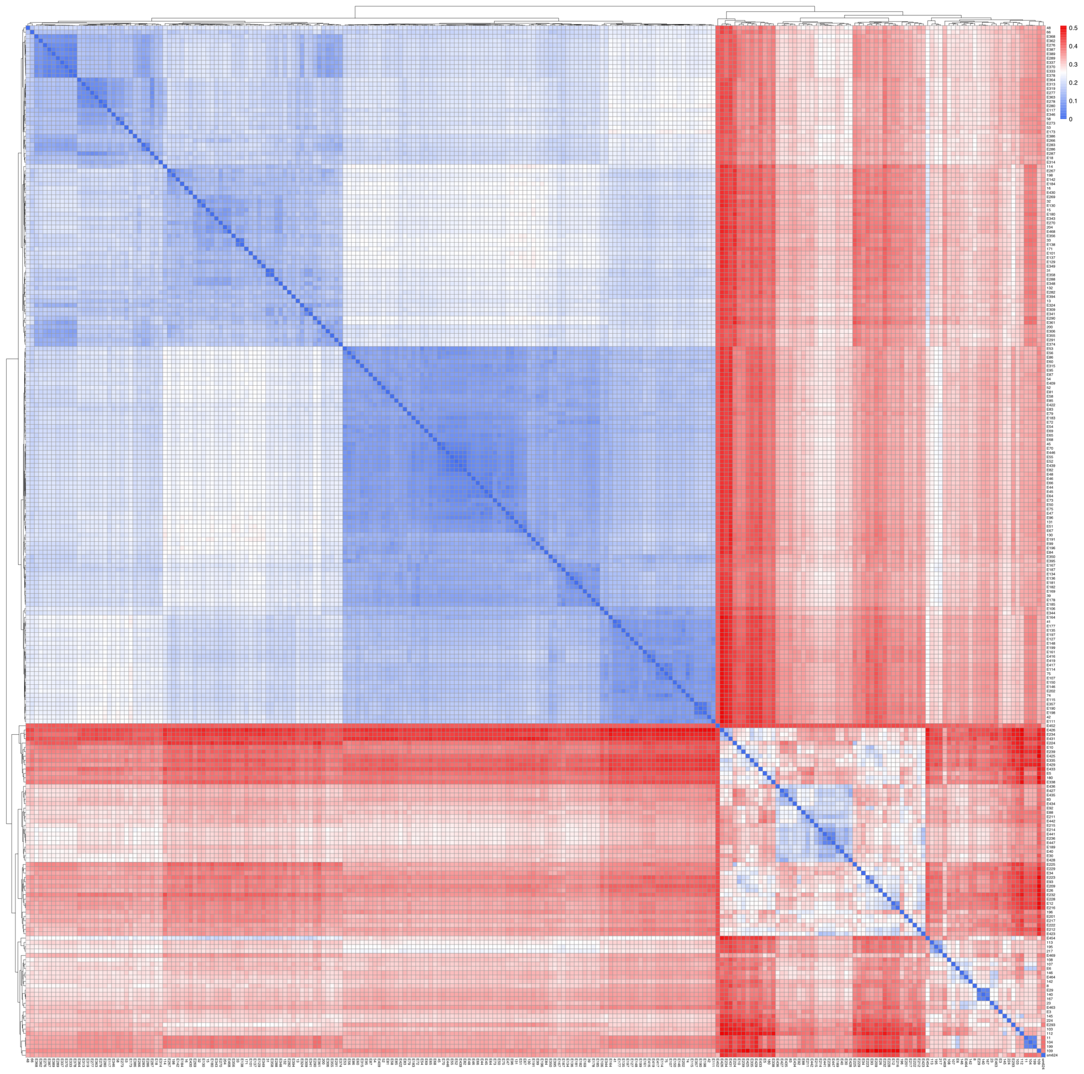

Figure S9. Heatmap of Sample Kinship Clustering, illustrating the kinship clustering among samples, providing a visual representation of their genetic relatedness.

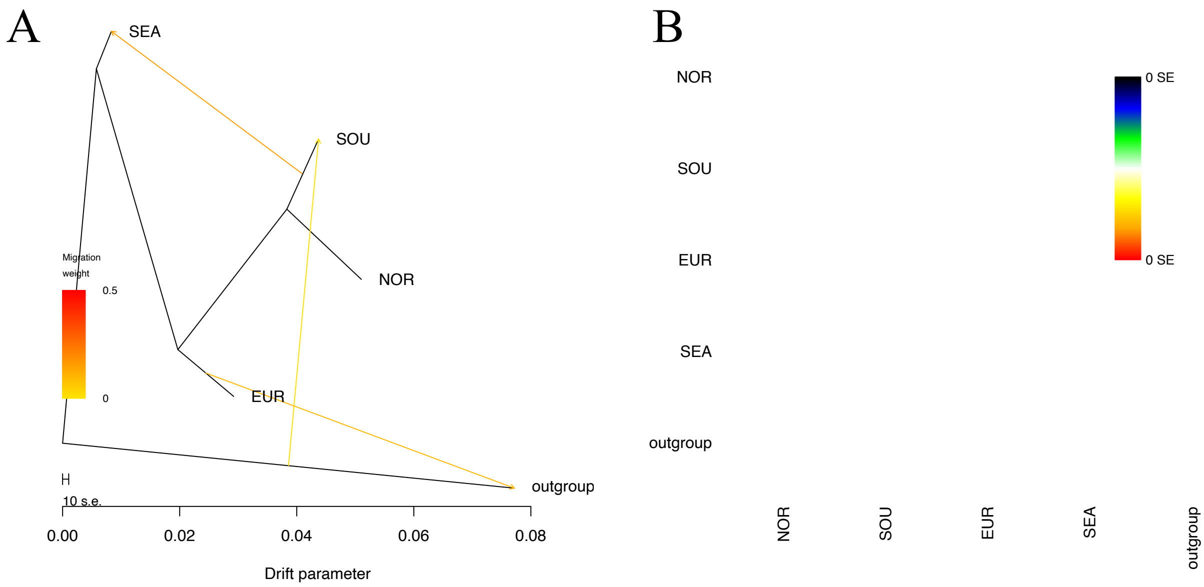

Figure S10. Inferred eggplant tree. A. Maximum Likelihood (ML)-tree inferred by TreeMix allowing three migration events.The ML-tree was constructed to analyze gene flow among eggplant populations from different regions. The populations are grouped into four main clusters: SEA (Southeast Asia), SOU (Southern China), NOR (Northern China), and EUR (Europe). Arrows indicate migration events inferred by TreeMix, with their direction representing the source and target populations. Migration arrows are colored according to their weight, with colors explained in the palette on the left. The scale bar represents ten times the average standard error (s.e.) of the entries in the sample covariance matrix. The x-axis shows the drift parameter, which represents the genetic drift among populations. **B.** Residual fit. Plotted is the residual fit from the ML-tree in panel A. The residual covariance between each pair of populations was divided by the average standard error across all pairs, and the scaled residuals are plotted in each cell. Colors are described in the palette on the right. Residuals above zero indicate populations that are more closely related to each other in the data than in the best-fit tree, suggesting possible admixture events. In this case, the residual fit shows no significant color changes, indicating that the ML-tree with three migration events provides a good fit to the covariance matrix of the populations.

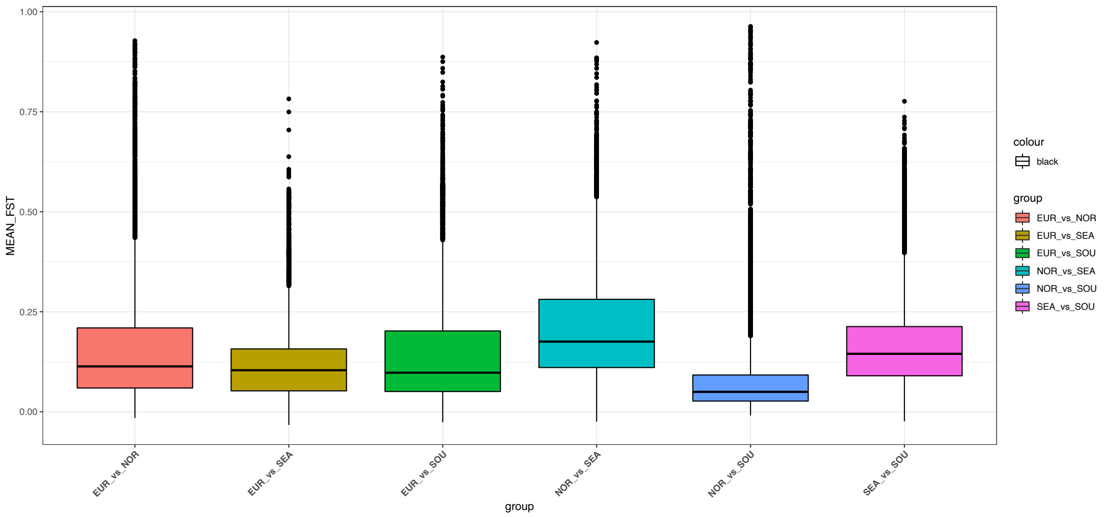

Figure S11. Box Plot of Genetic Differentiation Index (Fst) Between Four Populations. The figure illustrates a box plot representing the genetic differentiation index (Fst) between pairs of four distinct populations. The Fst index, ranging from 0 to 1, quantifies the degree of genetic divergence between two populations; a higher value indicates greater differentiation, with values exceeding 0.25 typically signifying significant divergence. The Fst calculations were performed using the VCFtools program, employing a sliding window approach with a window size of 3kb.

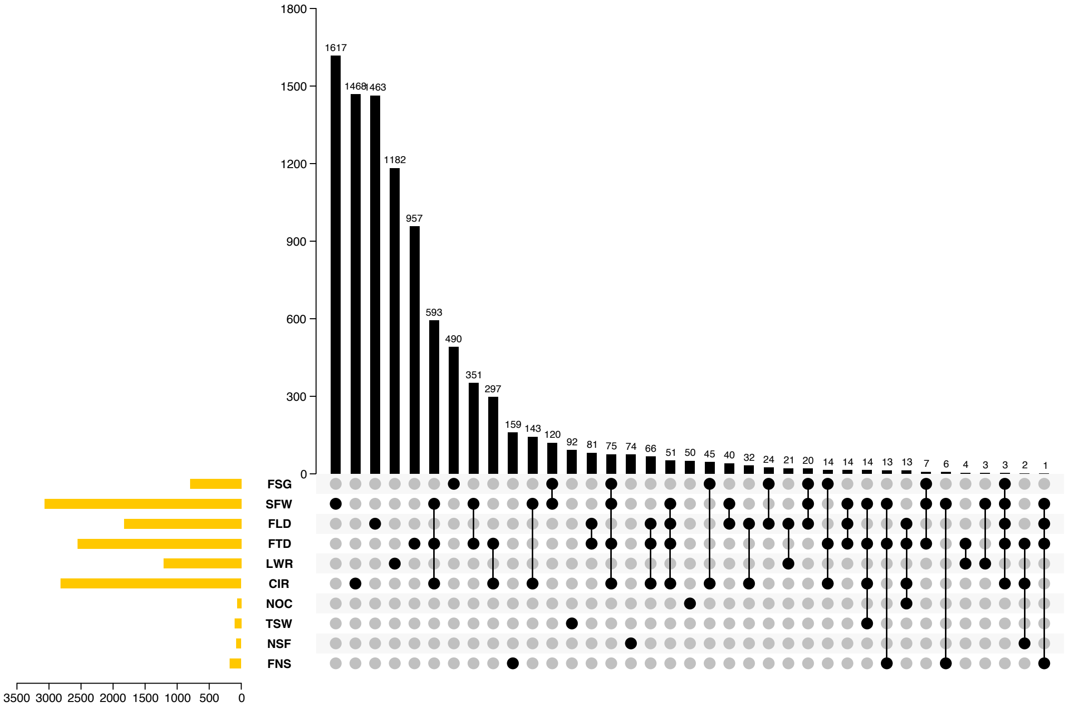

Figure S12. An upset plot of significantly associated genes for 10 important traits. The vertical axis represents the number of genes, while the horizontal axis displays the number of shared genes between different traits. The yellow bars indicate the total number of genes within the significantly associated loci regions for each trait.

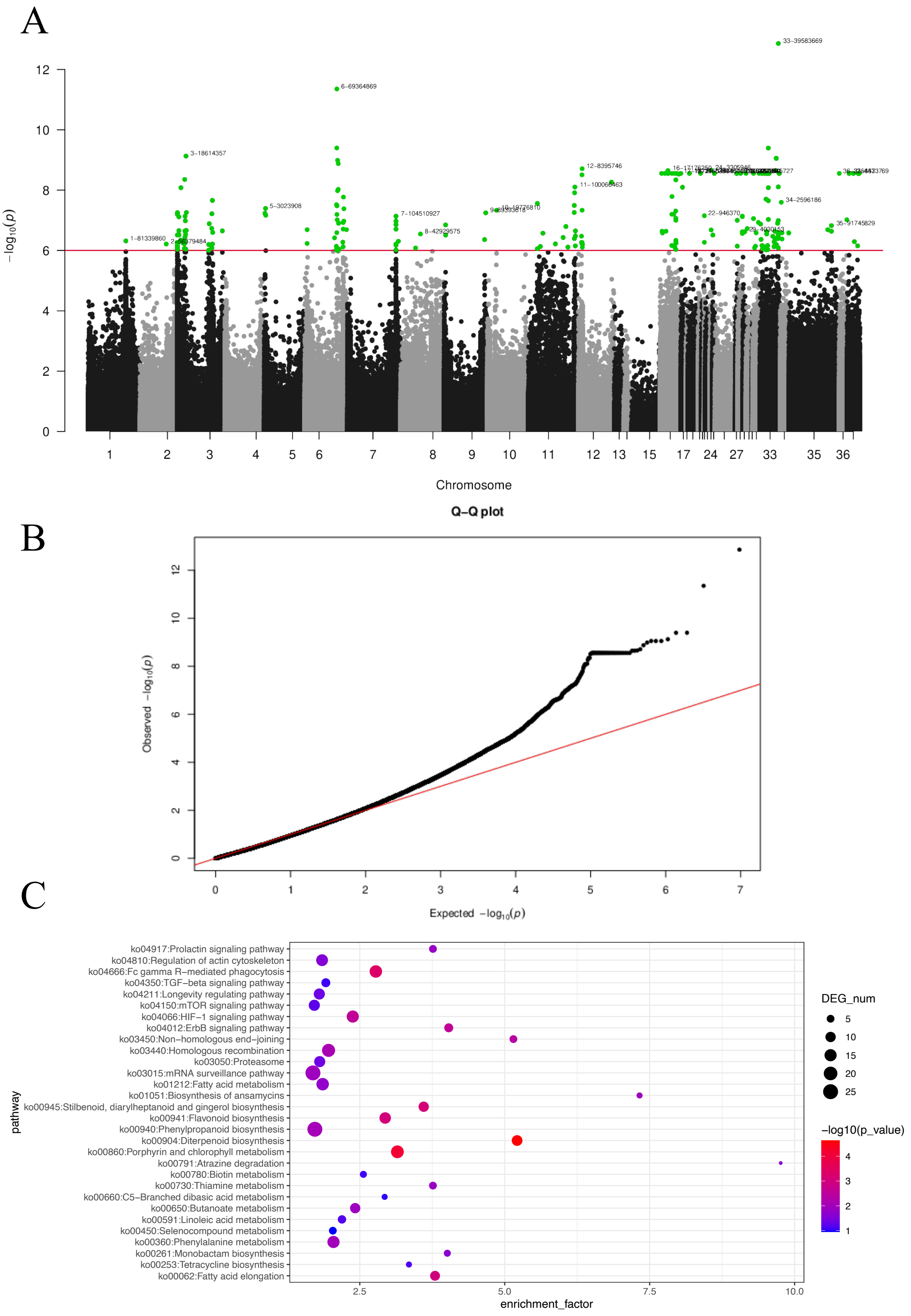

Figure S13. Genetic Association Study and Functional Enrichment Analysis of Ribbing Traits on SFW. A. Manhattan plot of the genome-wide association study (GWAS) for SFW. **B.** Q-Q plot of GWAS results for SFW, illustrating the observed versus expected distribution of p-values. **C.** KEGG pathway enrichment analysis for genes associated with SFW.

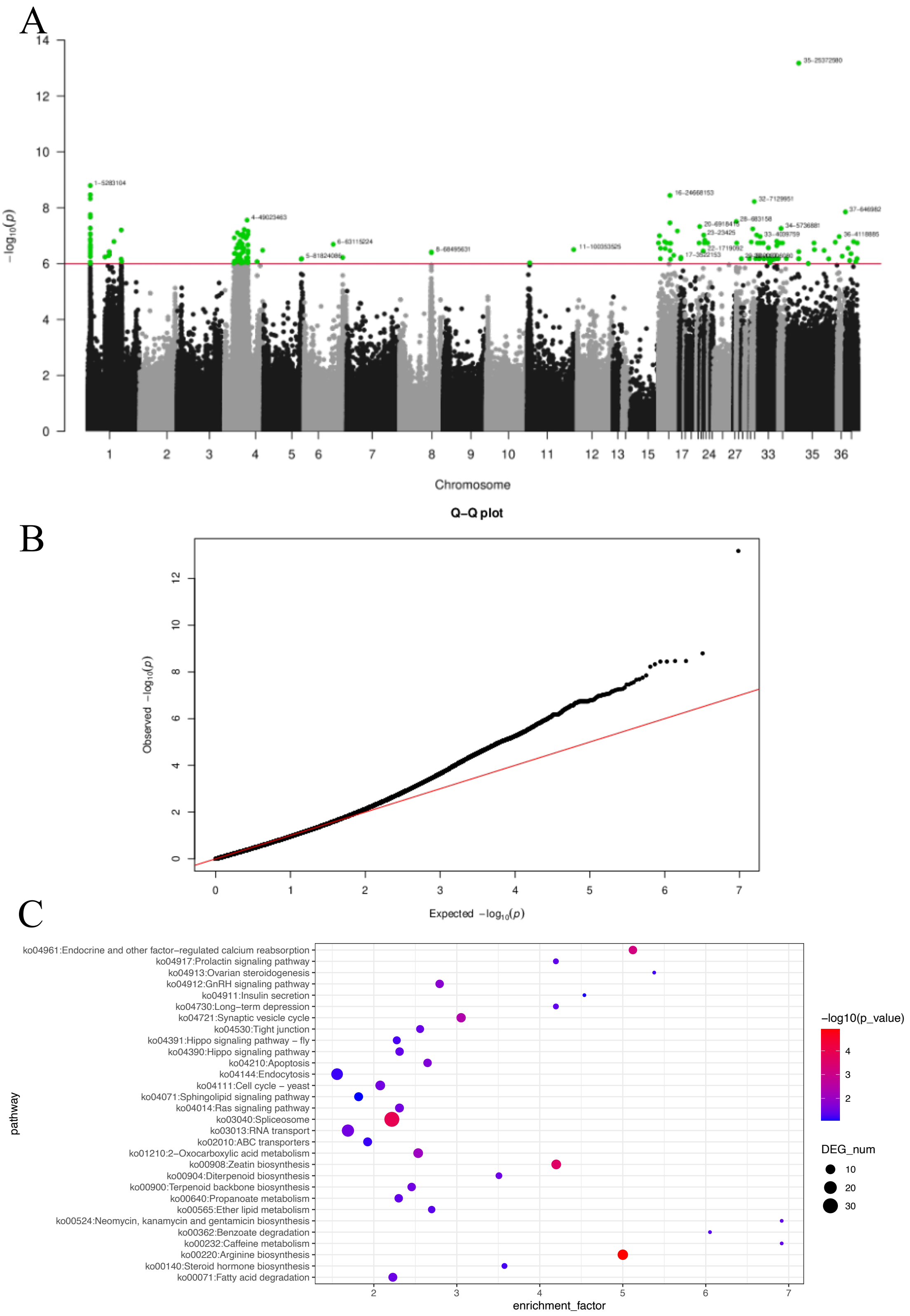

Figure S14. Genetic Association Study and Functional Enrichment Analysis of Ribbing Traits on FLD. A. Manhattan plot of the genome-wide association study (GWAS) for FLD. **B.** Q-Q plot of GWAS results for FLD, illustrating the observed versus expected distribution of p-values. **C.** KEGG pathway enrichment analysis for genes associated with FLD.

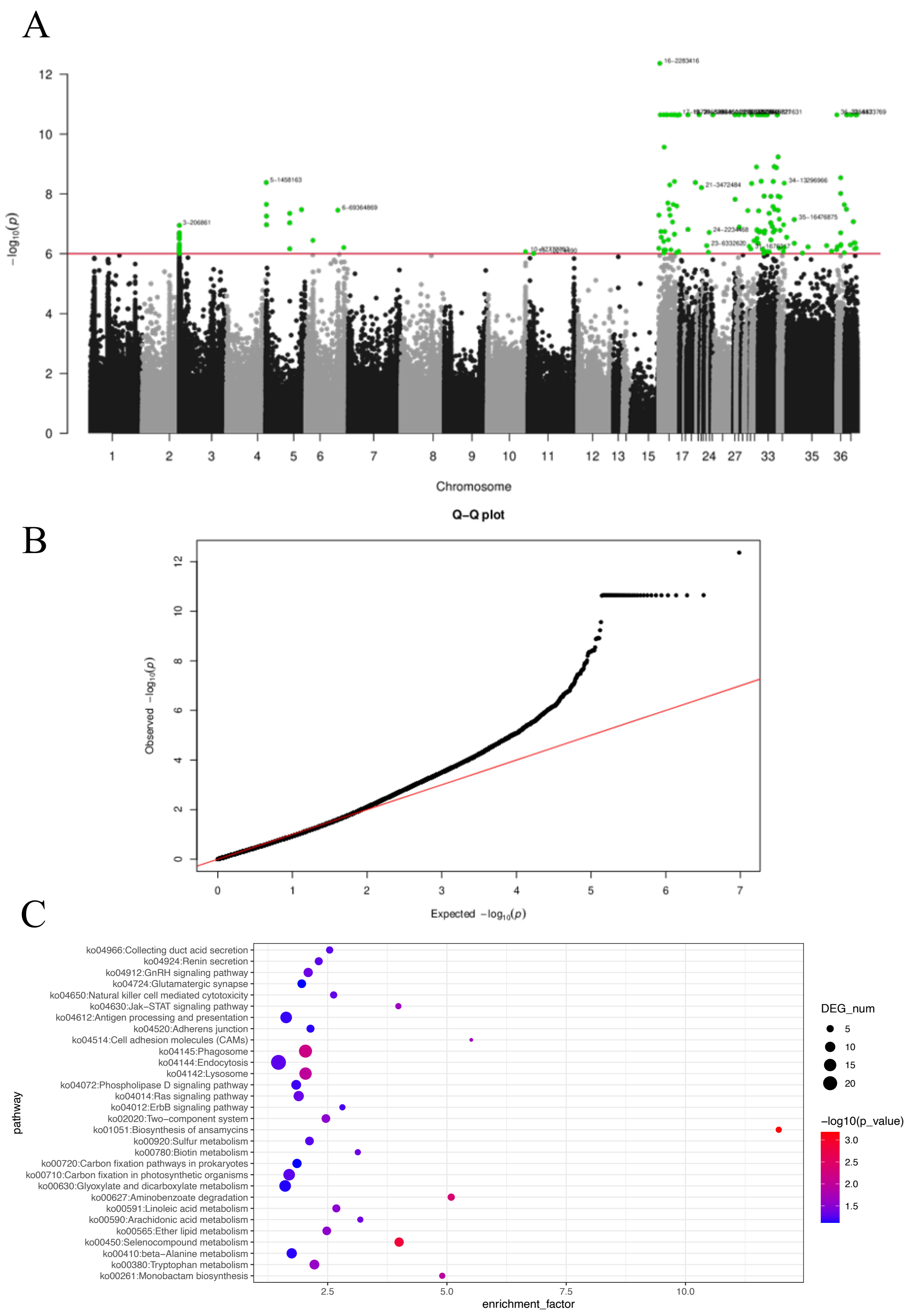

Figure S15. Genetic Association Study and Functional Enrichment Analysis of Ribbing Traits on FTD. A. Manhattan plot of the genome-wide association study (GWAS) for FTD. **B.** Q-Q plot of GWAS results for FTD, illustrating the observed versus expected distribution of p-values. **C.** KEGG pathway enrichment analysis for genes associated with FTD.

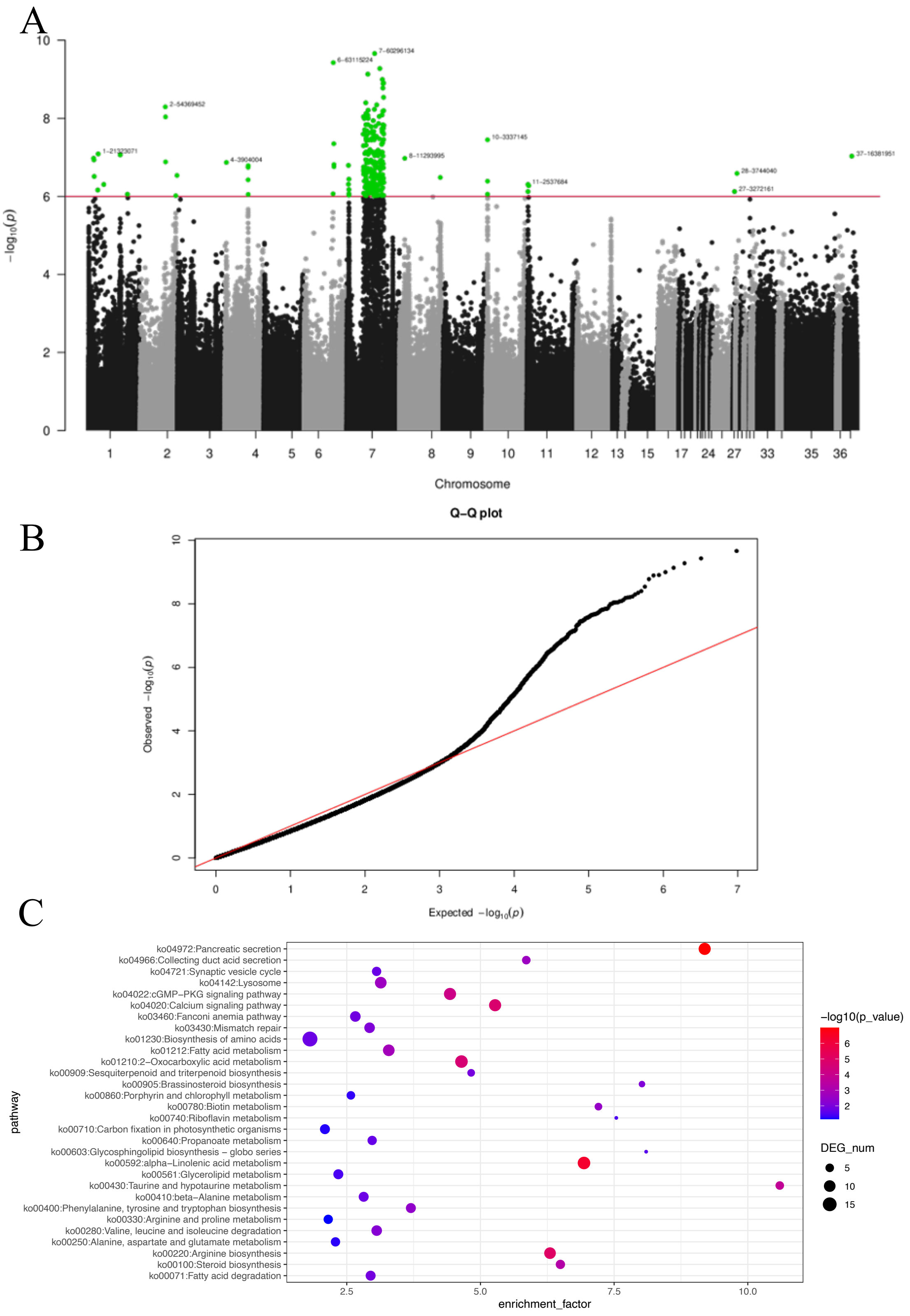

Figure S16. Genetic Association Study and Functional Enrichment Analysis of Ribbing Traits on LWR. A. Manhattan plot of the genome-wide association study (GWAS) for LWR. **B.** Q-Q plot of GWAS results for LWR, illustrating the observed versus expected distribution of p-values. **C.** KEGG pathway enrichment analysis for genes associated with LWR.

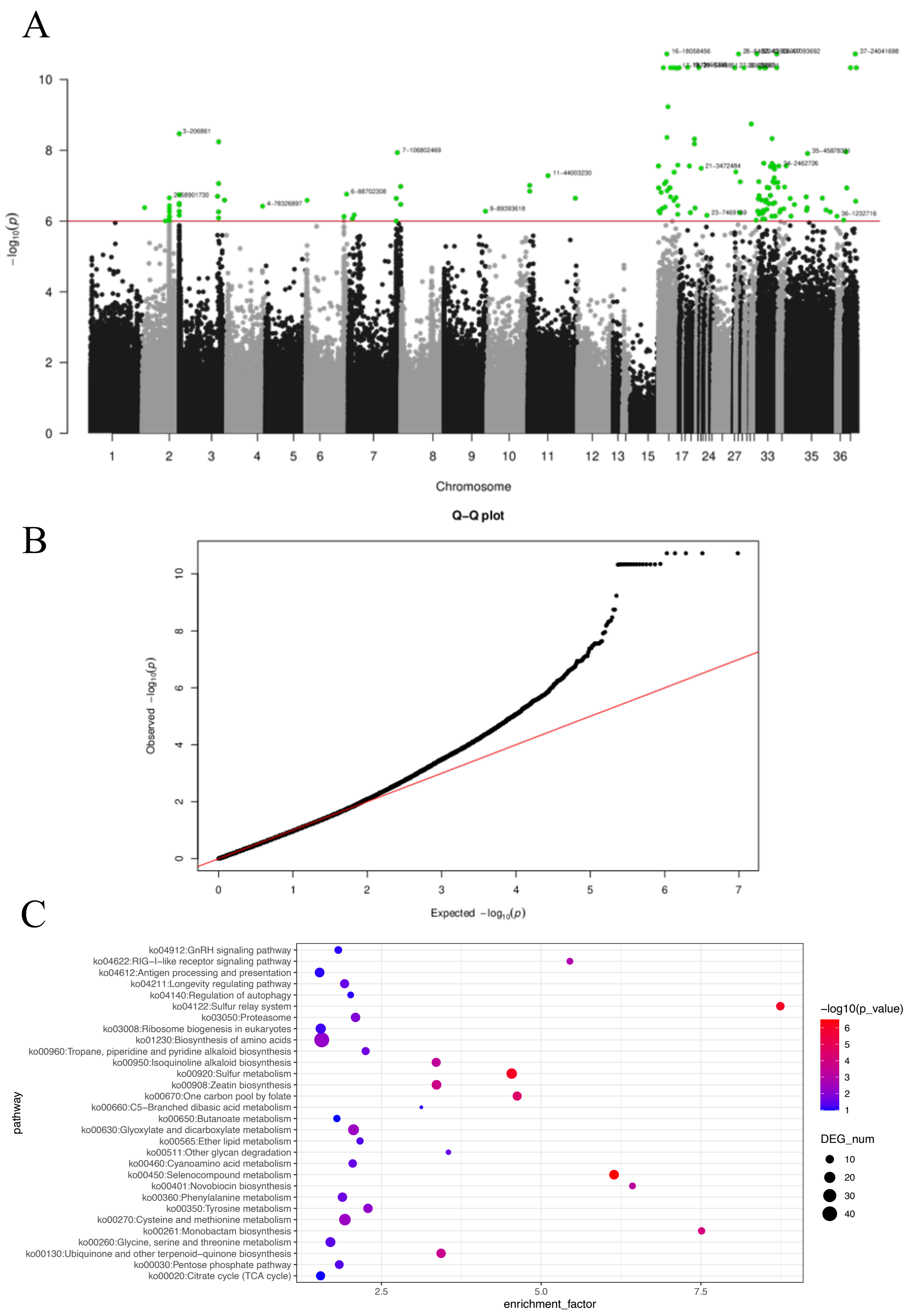

Figure S17. Genetic Association Study and Functional Enrichment Analysis of Ribbing Traits on CIR. A. Manhattan plot of the genome-wide association study (GWAS) for CIR. **B.** Q-Q plot of GWAS results for CIR, illustrating the observed versus expected distribution of p-values. **C.** KEGG pathway enrichment analysis for genes associated with CIR.

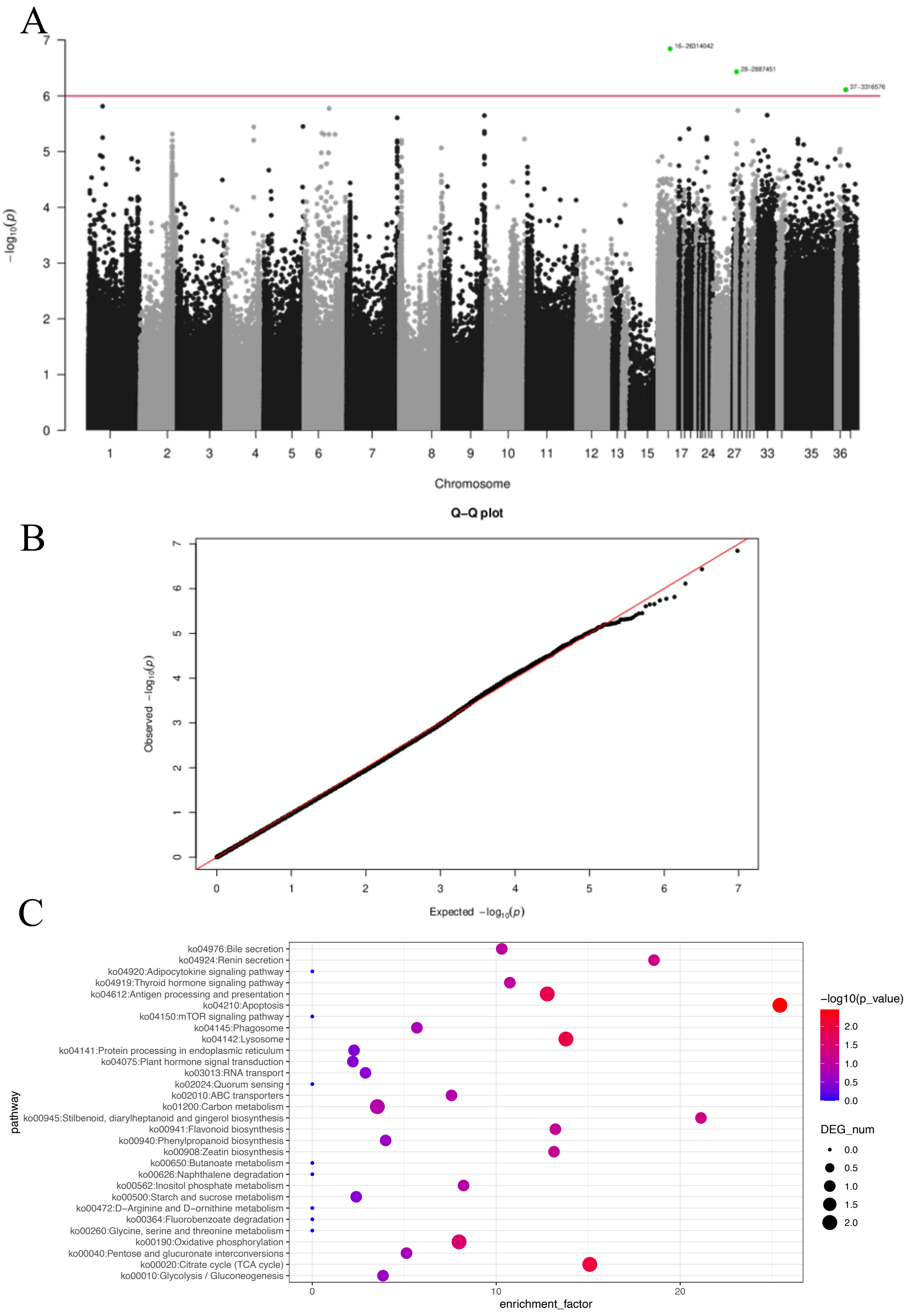

Figure S18. Genetic Association Study and Functional Enrichment Analysis of Ribbing Traits on NOC. A. Manhattan plot of the genome-wide association study (GWAS) for NOC. **B.** Q-Q plot of GWAS results for NOC, illustrating the observed versus expected distribution of p-values. **C.** KEGG pathway enrichment analysis for genes associated with NOC.

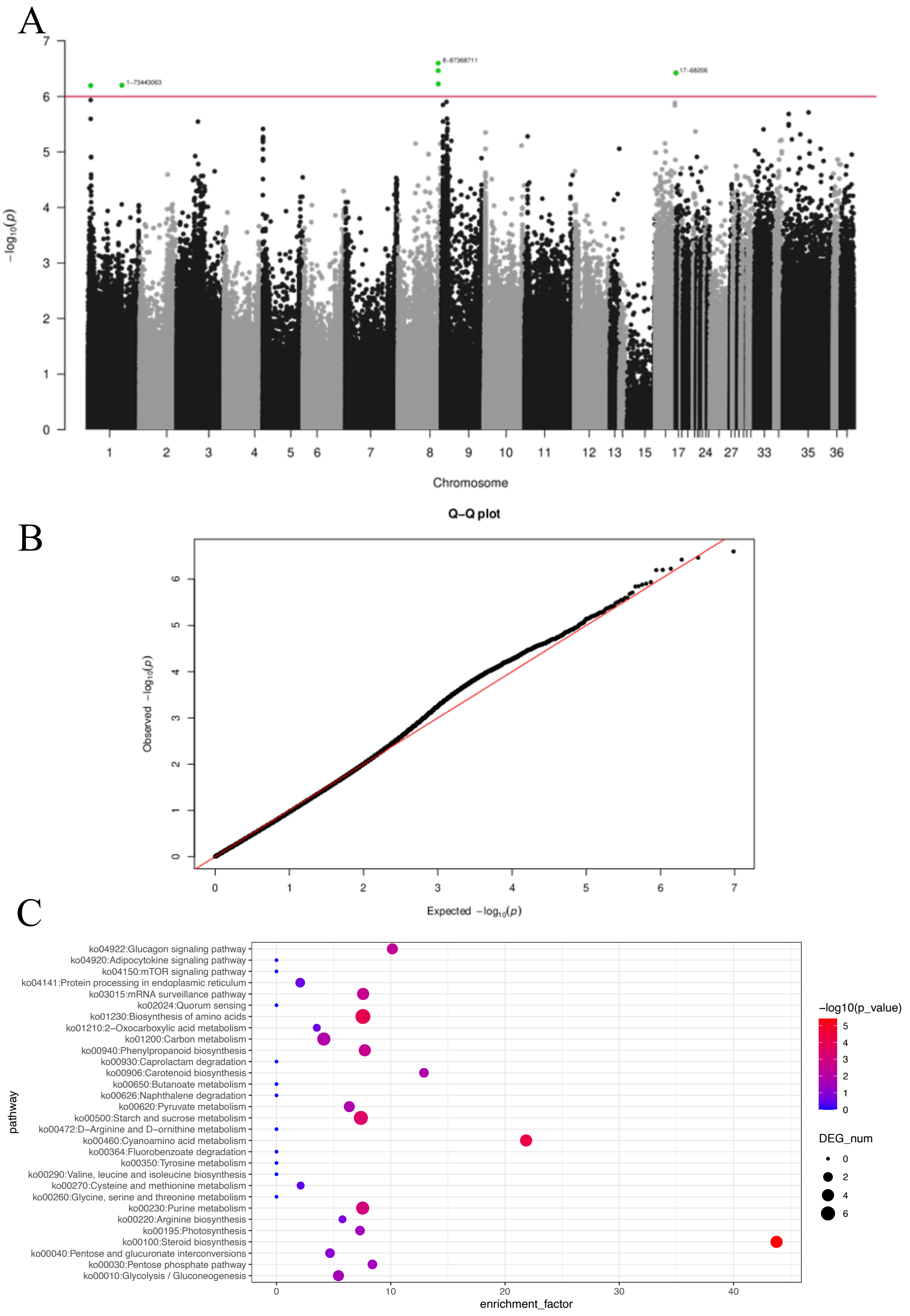

Figure S19. Genetic Association Study and Functional Enrichment Analysis of Ribbing Traits on TSW. A. Manhattan plot of the genome-wide association study (GWAS) for TSW. **B.** Q-Q plot of GWAS results for TSW, illustrating the observed versus expected distribution of p-values. **C.** KEGG pathway enrichment analysis for genes associated with TSW.

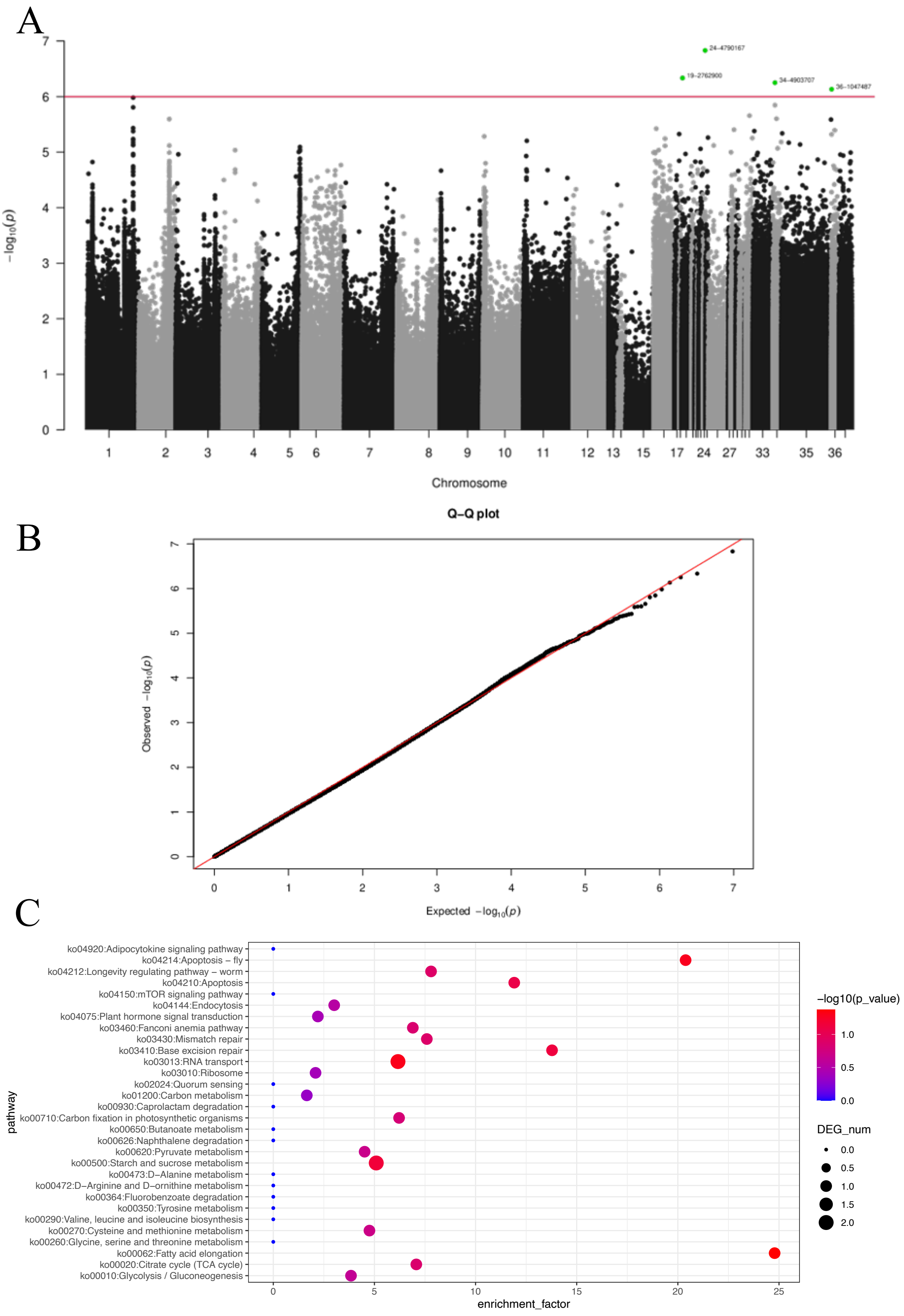

Figure S20. Genetic Association Study and Functional Enrichment Analysis of Ribbing Traits on NSF. A. Manhattan plot of the genome-wide association study (GWAS) for NSF. **B.** Q-Q plot of GWAS results for NSF, illustrating the observed versus expected distribution of p-values. **C.** KEGG pathway enrichment analysis for genes associated with NSF.

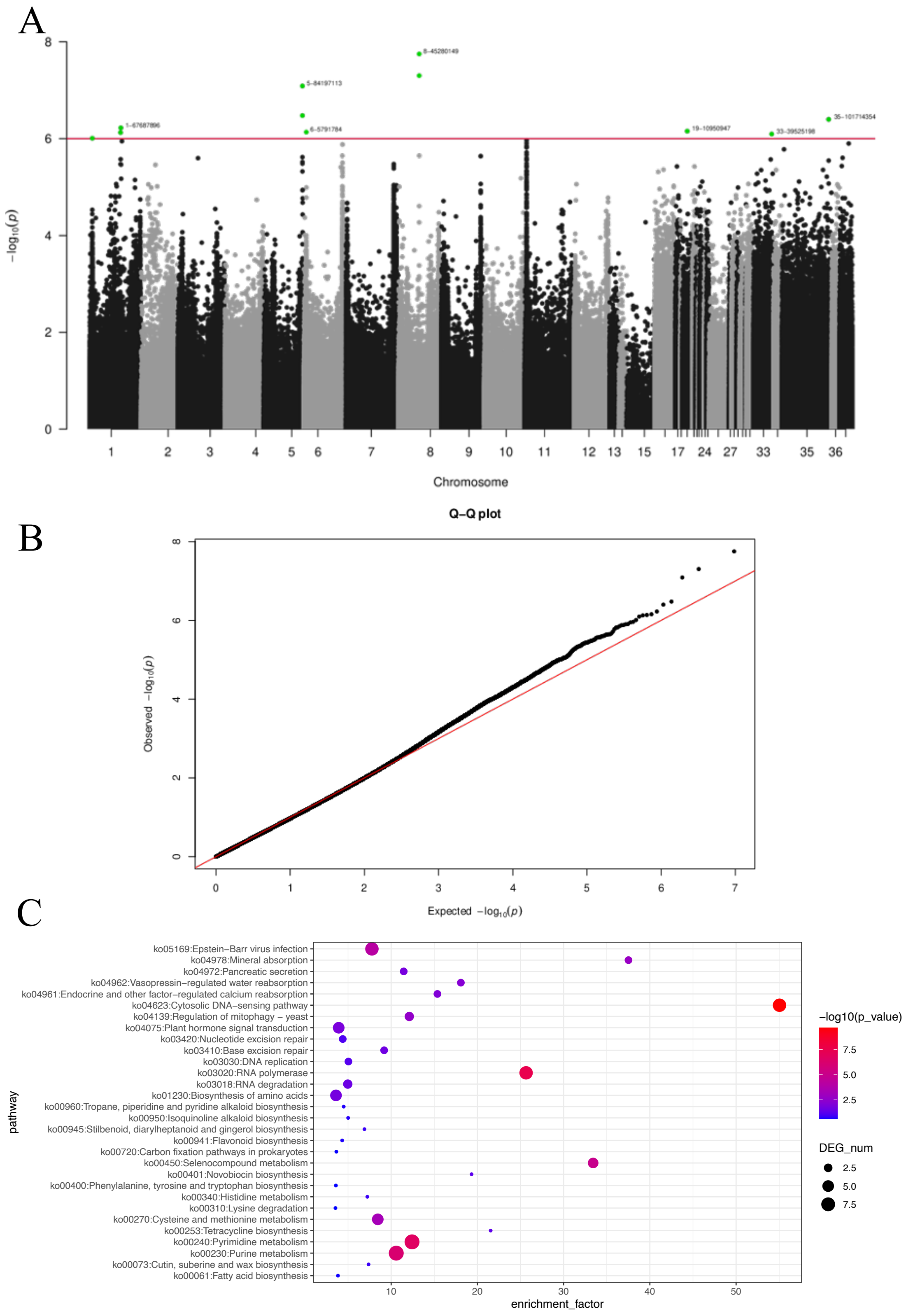

Figure S21. Genetic Association Study and Functional Enrichment Analysis of Ribbing Traits on FNS. A. Manhattan plot of the genome-wide association study (GWAS) for FNS. **B.** Q-Q plot of GWAS results for FNS, illustrating the observed versus expected distribution of p-values. **C.** KEGG pathway enrichment analysis for genes associated with FNS.
